# Shaping of developmental gradients through selection on multiple loci in *Antirrhinum*

**DOI:** 10.1126/sciadv.adx2011

**Published:** 2026-07-17

**Authors:** Desmond Bradley, Louis Boell, Daniel Richardson, Lucy Copsey, Annabel Whibley, Ting Xu, Yu’e Zhang, Yongbiao Xue, David Field, Enrico Coen

**Affiliations:** ^1^ John Innes Centre, Norwich, UK.; ^2^ School of Biological Sciences, University of Auckland, Auckland, New Zealand.; ^3^ Institute of Genetics and Developmental Biology, Chinese Academy of Sciences, Beijing, China.; ^4^ Xianghu Laboratory, Hangzhou 311231, China.; ^5^ Institute of Science and Technology Austria, Klosterneuburg, Austria.; ^6^ Applied BioSciences, Macquarie University, Sydney, Australia.

## Abstract

Development depends on precise shaping of molecular gradients, but how natural selection acts to establish precision is unknown. Here, we analyze genes that control differences in the gradient of yellow flower color between two varieties of snapdragon (*Antirrhinum*). We show that these differences depend, in part, on cis-regulatory variation in the pigment biosynthetic gene, *FLAVIA* (*FLA*). *FLA* interacts multiplicatively with three other loci, one of which is a trans-acting regulator of *FLA*, to further shape the yellow gradient. All the loci exhibit clines at a hybrid zone, with widths that correlate with phenotypic effect, showing how selection can hone gradient shape with remarkable precision by acting on cis and trans variation at multiple loci.

## Teaser

Evolutionary sharpening of flower color patterns with molecular paintbrushes.

## INTRODUCTION

Pattern formation is central to development, from establishment of regional identities in early embryos (*1*), to the generation of coloration patterns in adults (*2*). A key component of pattern formation is the shaping of gradients, achieved with great reproducibility and precision (*3*–*5*). However, it is unclear how natural selection acts to tune gradient properties. Hybrid zones, where species or varieties undergo prolonged gene exchange (*6*, *7*), provide an opportunity to address this problem. Loci under selection can be recognized through steep clines in allele frequency. Some of the identified loci under selection modify color patterns, such as the aposematic markings on butterfly wings (*8*, *9*) or floral guides (*10*). Studying how such loci interact can reveal how color gradients are tuned by selection. Here, we apply this approach to flower color variation between two varieties of snapdragon, *Antirrhinum majus*.

## RESULTS AND DISCUSSION

**Fig. 1.**
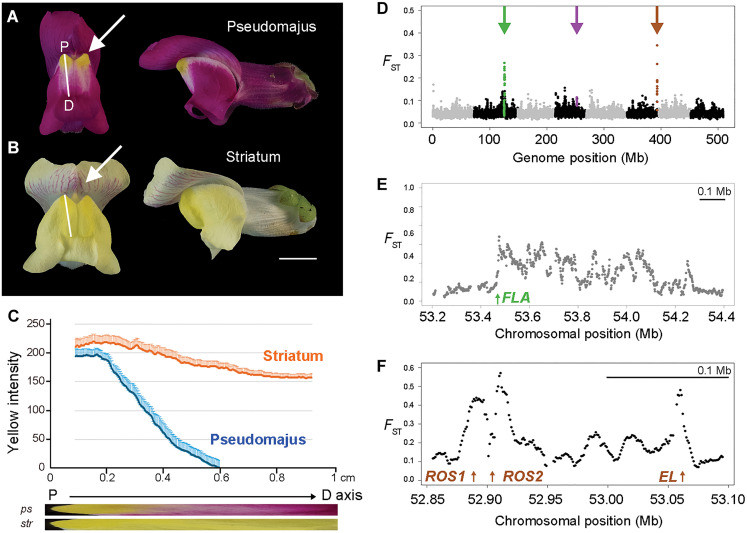
Flower color phenotypes and *F*_ST_ genome scans. (**A**) *A.m.m*. var. *pseudomajus* flower in face (left) and side (right) views. Arrow indicates yellow highlight (focus) at the bee entry point. White line shows the transect used to measure the yellow gradient, from the proximal (P) to distal (D) edge of the face. (**B**) *A.m.m.* var. *striatum* flower, with arrow pointing to magenta veins in the upper petals. Scale bar, 1 cm. (**C**) Yellow intensity along transects as measured by excess green reflectance over blue for striatum (orange line) and pseudomajus (blue line). For each flower, transect lengths were scaled to 1 cm and reflectance averaged for 10 individuals. Transect photographs are illustrated below the graph. (**D**) Whole-genome *F*_ST_ scan between a population of *A.m.m.* var. *striatum* (YP1) and *A.m.m.* var. *pseudomajus* (MP4), summarized in windows of 50 kb with 25-kb overlaps. Chromosomes 1 to 8 are shown in alternating black and gray. Purple arrow on Chr4 points to the *SULF* locus, brown arrow on chr 6 to the *ROS/EL* loci, and green arrow to a novel island on Chr2. The peak at the start of Chr1 corresponds to *CRE* (*22*) and the peak on Chr5 to *RUB* (*21*). (**E**) Close-up of *F*_ST_ island on Chr2 (10-kb windows, with 9-kb overlaps), giving a *F*_ST_ island of 789 kb based on a 0.2 threshold. (**F**) Close-up of *F*_ST_ island on Chr6, with *ROS* and *EL* loci denoted by arrows, magnified 4.8 times more than (E) for size comparison. The *ROS* island is 69 kb long and *EL* 29 kb based on a 0.2 threshold.

The *Antirrhinum* genus comprises about 25 species primarily distributed throughout the western Mediterranean (*11*). The species exhibit diverse flower color patterns. These patterns serve as guides for flower entry by pollinators, mainly bees. Two varieties of *A. majus* subspecies *majus*, *A.m.m.* var. *pseudomajus* and *A.m.m.* var. *striatum* (*12*), live in close proximity and have complementary flower color patterns. *A.m.m.* var. *pseudomajus* has a yellow highlight (arrowed in Fig. 1A), against a contrasting magenta background, whereas *A.m.m.* var. *striatum* has a magenta highlight (arrowed in Fig. 1B) against a yellow background. Yellow and magenta excite different components of the bee visual system, which is based on green-blue-ultraviolet receptors rather than red-green-blue of vertebrates. Yellow excites green receptors, whereas magenta excites blue (ultraviolet reflectance is low for flowers of both varieties (*13*)).

### Yellow gradient steepness varies between varieties

The yellow color forms a gradient on the lower (ventral) petal lobe, with the intensity of yellow declining from the point of bee entry toward the edge of the petal. The gradient can be quantified by plotting excess of green over blue reflectance along a transect (white lines in Fig. 1, A and B). *A.m.m.* var. *pseudomajus* shows a steep decline in yellow, whereas in *A.m.m.* var. *striatum*, the yellow gradient is much shallower (Fig. 1C). Comparison of four different sets of independent populations gave similar gradient patterns (fig. S1). The difference in yellow gradient steepness between varieties is likely related to the distribution of magenta. In *A.m.m.* var. *pseudomajus*, the steep gradient reduces overlap of yellow with magenta in distal regions of the petal and thus mixing of complementary colors, which would reduce visual contrast. In *A.m.m.* var. *striatum*, magenta is absent in the ventral lobe, allowing extension of yellow into the distal regions of the petal without pigment mixing.

### An island of high relative divergence is located on chromosome 2

Selection on three major loci, *ROSEA* (*ROS*), *ELUTA* (*EL*), and *SULFUREA* (*SULF*), contributes to maintaining color pattern differences between *A.m.m.* var. *pseudomajus* and *A.m.m.* var. *striatum* across a hybrid zone (*10*, *14*, *15*). In contrast to most of the genome, which shows little differentiation across the hybrid zone, all three loci exhibit steep clines in allele frequency, a signature for barriers to gene flow. *ROS* and *EL* encode *MYB* transcription factors that regulate magenta pigment (anthocyanin) biosynthesis (*10*, *16*, *17*), whereas *SULF* encodes small RNAs that restrict the distribution of yellow pigment (aurone) by targeting a biosynthetic gene (*15*).

*ROS* and *EL* are tightly linked (0.5 centimorgans apart) and exhibit peaks or islands of high relative divergence (*F*_ST_) in genome comparisons between populations either side of the hybrid zone (brown arrow, Fig. 1D) (*10*). *SULF* (purple arrow) does not exhibit a strong *F*_ST_ peak, most likely because of polymorphic deletions near the locus, which reduce sequence depth and prevent reliable estimates of *F*_ST_ (*15*).

In addition to the island of high *F*_ST_ at ROS EL, we observed a second high peak of *F*_ST_ (green arrow, Fig. 1D), located on chromosome 2. The chromosome 2 island did not correspond to a known genetic locus responsible for trait differences between *A.m.m.* var. *striatum* and *A.m.m.* var. *pseudomajus* and extended over a greater region than that encompassing the *ROS* and *EL F*_ST_ peaks (*10*) (Fig. 1, E and F). To determine whether the greater size of the chromosome 2 island was due to low recombination rates, we genotyped an F2 of *A.m.m.* var. *striatum* and *A.m.m.* var. *pseudomajus* for several single-nucleotide polymorphisms (SNPs) in and around the chromosome 2 island (fig. S2A). The recombination rate around the chromosome 2 island was about 0.1 cM/Mb, 30 times lower than 3 cM/Mb measured for *ROS/EL* region (*10*). Similar low recombination rates were observed when either *A.m.m.* var. *striatum* or *A.m.m.* var. *pseudomajus* varieties were crossed to cultivated *A.m.majus*, indicating that a chromosome inversion between the varieties was not responsible (fig. S2, B and C).The low recombination rate most likely reflected location of the chromosome 2 island in a pericentromeric region (*18*).

### The chromosome 2 island harbors a novel flower color locus, *FLA*

One explanation for the chromosome 2 island is that it harbors a previously unidentified flower-color locus under selection. To test this hypothesis, we analyzed 160 plants from a F2 of *A.m.m.* var. *striatum* crossed to *A.m.m.* var. *pseudomajus*. We genotyped for a SNP within the chromosome 2 island (SNP2A) and SNPs for known color loci *ROS*, *EL*, and *SULF*. Flowers for each *ROS EL SULF* genotype were ranked visually according to the spread of magenta or yellow flower color and divided into high (top 50%) and low (bottom 50%) groups (fig. S3). All rankings were made before knowledge of genotypes and repeated three times independently, by different researchers.

**Fig. 2.**
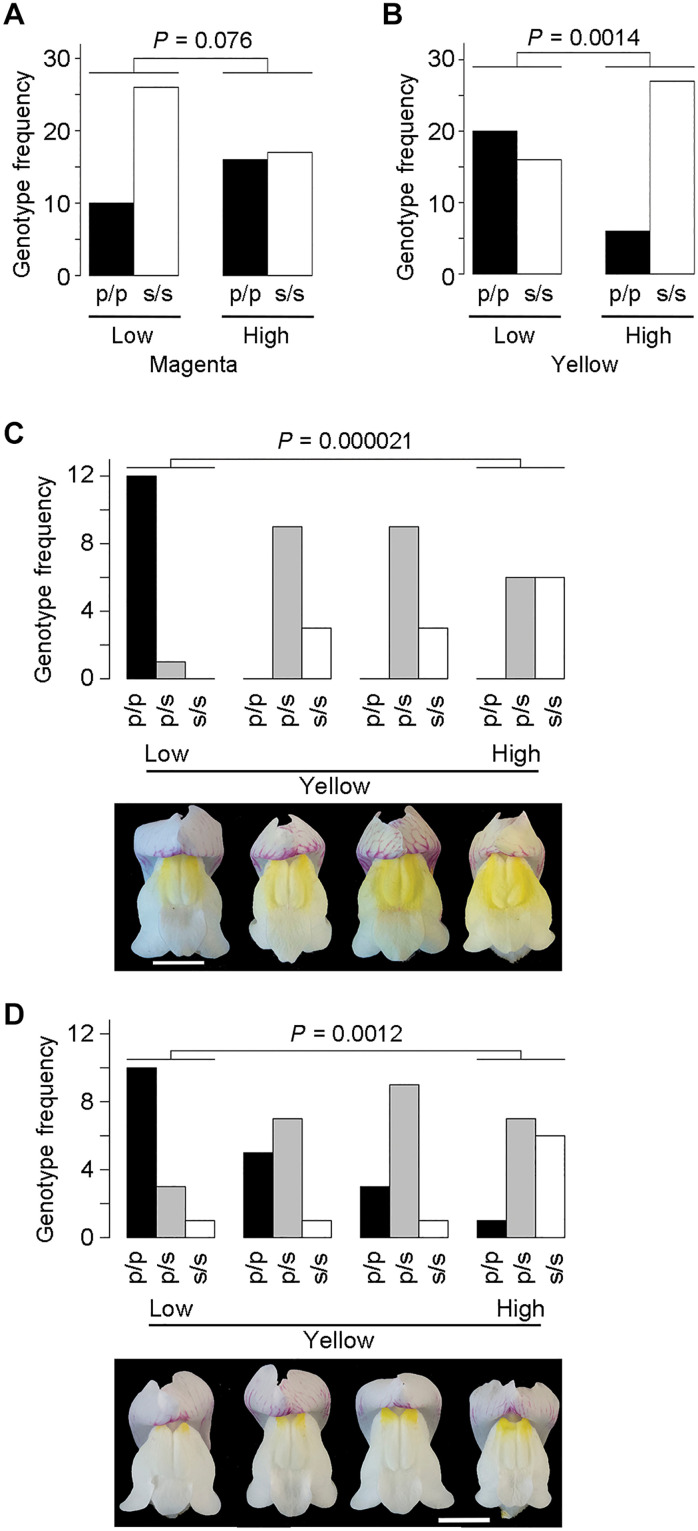
Identification of *FLA*. (**A**) Ranking results from an F2 population (*n* = 69) of *A.m.m.* var. *pseudomajus* (*p*) x *A.m.m.* var. *striatum* (*s*) ranked for extent of petal lobe magenta and genotyped for SNP2A. Individuals were grouped and ranked separately according to *ROS*, *EL*, and *SULF* genetic backgrounds, and results were aggregated across all genotypic classes. The low and high magenta categories have no significant difference in frequencies of p/p and s/s homozygotes (*P* values calculated using a contingency χ^2^ test between the low and high quartiles). (**B**) Ranking the same population for extent of yellow shows striatum homozygotes significantly enriched in the high yellow category. (**C**) A *ros^s^ El^s^/ros^s^ El^s^ sulf^s^/sulf^s^* population genotyped for SNP2A and ranked for the extent of yellow. The lowest yellow quartile is significantly enriched for p/p homozygotes. Examples of flowers from the middle of each quartile are shown below. Scale bar, 1 cm. (**D**) A *ros^s^ El^s^/ros^s^ El^s^ SULF^p^/SULF^p^* population ranked for their extent of yellow. The lowest yellow quartile is significantly enriched for p/p homozygotes. Scale bar, 1 cm. Yellow rankings in (B) and (C) were done in triplicate by three independent observers and all gave similar significant *P* values (fig. S3).

No significant differences in SNP2A homozygote frequencies were observed between high and low magenta groups (Fig. 2A). However, an excess of striatum homozygotes and deficit of the pseudomajus homozygotes was observed in the high versus low yellow group (Fig. 2B). This difference was confirmed by ranking F4 populations segregating for SNP2A in a *ros^s^ EL^s^ sulf^s^* background into quartiles (Fig. 2C and fig. S4, allele superscripts refer to origin, *s* for striatum and *p* for pseudomajus). Plants in the lowest yellow quartile were mainly homozygous for the pseudomajus SNP2A allele, whereas those in the highest yellow quartile were homozygous or heterozygous for the striatum SNP2A allele, indicating that the striatum allele was dominant. Similar results were obtained in a *ros^s^ EL^s^ SULF^p^* background, although there was greater overlap between quartiles, most likely because reduction in yellow caused by *SULF^p^* made differences harder to discern (Fig. 2D and fig. S5). Thus, the chromosome 2 island harbors a novel yellow flower color locus, hereafter referred to as *FLAVIA* (*FLA*), with the striatum allele denoted as *FLA^s^* and pseudomajus allele *fla^P^.*

### Both cis and trans variation controls the gradient of *FLA* expression

**Fig. 3.**
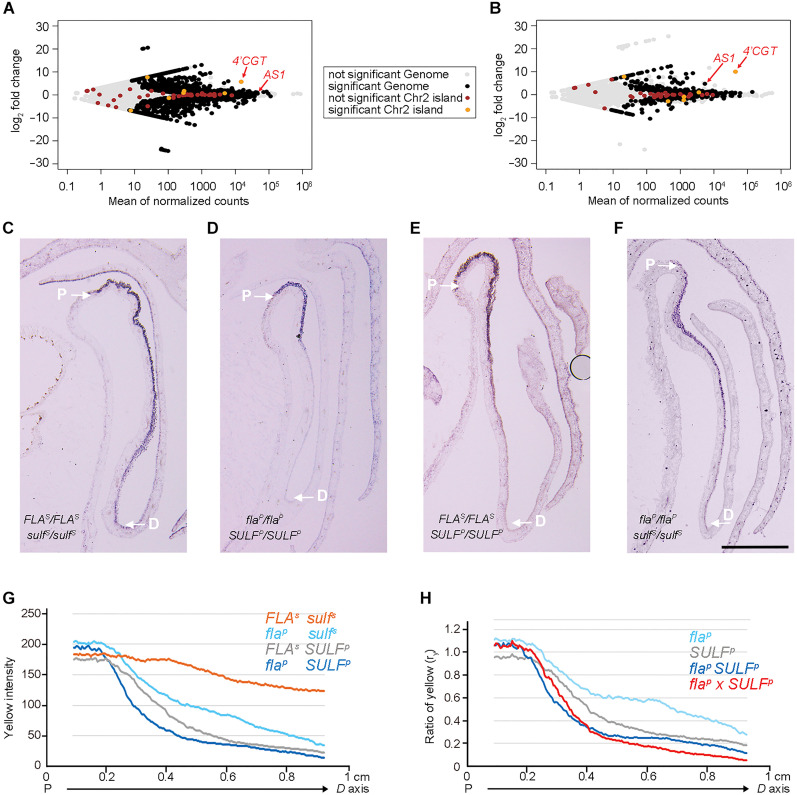
*FLA* likely encodes *4*′*CGT* and interacts multiplicatively with *SULF*. (**A**) Log fold change in transcripts from the chromosome 2 island between *A.m.m.* var. *striatum* and *A.m.m.* var. *pseudomajus. 4*′*CGT* transcript indicated. Transcripts showing differential expression between the pseudomajus and striatum samples (DESeq2 adjusted *P* value <0.01) are highlighted for the genome in black, insignificant in gray. Significant DE transcripts from the Chr2 island are in orange, insignificant island transcripts in brown. (**B**) As in (A) except that the chromosome islands of *A.m.m.* var. *pseudomajus* and *A.m.m.* var. *striatum* were introgressed into a common *sulf^s^/sulf^s^* background. (**C**) RNA in situ showing *FLA^s^* expression (dark purple color) in *A.m.m.* var. *striatum*. The proximal (P) and distal (D) positions of the petal face used for transect color profiles are indicated. (**D**) *fla^p^* expression in *A.m.m.* var. *pseudomajus*. (**E**) *FLA^s^* expression in *SULF^p^*. (**F**) *fla^p^* expression in *sulf^s^* (**G**) YPs for different combinations of *FLA* and *SULF* alleles (all homozygous), with *FLA^s^ sulf^s^* (orange) corresponding to the *A.m.m.* var. *striatum* genotype, and *fla^p^ SULF^p^* (dark blue) corresponding to the *A.m.m.* var. *pseudomajus* genotype. Recombinants are in gray or cyan. (**H**) Ratio of yellow intensity, *r_y_*, determined by dividing values at each point of the transect in (A) by the value for *sulf^s^ FLA^s^* at that point. The *fla^p^* x *SULF^p^* predicted *r_y_* (red line) was calculated by multiplying their *r_y_* values at each point. Scale bar for all in situ sections is 1 mm and show as black line in (F).

To identify the *FLA* gene, we compared RNA sequencing (RNA-seq) data from flower buds of *A.m.m.* var. *striatum* and *A.m.m.* var. *pseudomajus* for transcripts mapping to the chromosome 2 island (Fig. 3A). The strongest expression difference within the chromosome 2 island (53-fold greater in *A.m.m.* var. *striatum*) was in transcripts encoding chalcone 4′-O-glucosyltransferase (4′CGT), an enzyme required for aurone (yellow pigment) biosynthesis. 4′CGT is the target of *SULF*, and the low expression in *A.m.m.* var. *pseudomajus* could therefore be due to repression by *SULF^p^* in trans. To test this possibility, we introgressed the chromosome 2 island from each subspecies into a common *sulf^s^/sulf*^s^ background. The striatum 4′CGT allele was still expressed at a higher level than that of pseudomajus (Fig. 3B), indicating that *FLA* likely encodes 4′CGT. RNA in situ hybridizations (Fig. 3, C and D) showed that *FLA* was expressed in a shallow proximodistal gradient in *A.m.m.* var. *striatum* (*FLA^s^ sulf^s^*) but a steep gradient in *A.m.m.* var. *pseudomajus* (*fla^p^ SULF^p^*). To resolve cis and trans effects, we generated recombinant genotypes *FLA^s^ SULF^p^* and *fla^p^ sulf^s^.* These exhibited intermediate gradients in *FLA* expression compared to the parental genotypes (Fig. 3, E and F). The results show that in either *SULF^p^* or *sulf^s^* backgrounds, *fla^p^* expression is less spread than *FLA^s^* along the proximodistal axis indicating that *fla^p^* acts in cis to restrict expression and sharpen the gradient. In either *FLA^s^* or *fla^p^* backgrounds, *FLA* expression is more restricted by *SULF^p^* than *sulf^s^*, showing that *SULF^p^* acts in trans to sharpen the gradient for both *FLA* alleles. Thus, relative to their striatum alleles, *fla^p^* acts in cis to reduce *FLA* expression, whereas *SULF^p^* acts in trans, via small RNAs, to inhibit *FLA.*

### *FLA* and *SULF* interact multiplicatively to shape the yellow gradient

To understand how *FLA* and *SULF* alleles interact to control the gradient in yellow flower pigment, we compared yellow intensity in transects of the ventral petal lobe, for genotypes in a low magenta (*ros EL*) background (Fig. 3G). Flowers with the *A.m.m.* var. *striatum* genotype (*FLA^s^ sulf^s^*) had a shallow yellow gradient (orange line). Introducing either *fla^p^* (cyan line, *fla^p^ sulf^s^*) or *SULF^p^* (gray line, *FLA^s^ SULF^p^*), steepened the gradient. Introducing both *fla^p^* and *SULF^p^* (dark blue line, *fla^p^ SULF^p^*), gave an even steeper gradient.

The reduction in pigmentation caused by pseudomajus alleles could be quantified by calculating the ratio, *r_y_*, of yellow intensity of flowers carrying these alleles to that in the striatum genotype, for each position along the transect*.* Both *fla^p^* and *SULF^p^* gave a downward proximodistal gradient in *r_y_* showing that pseudomajus alleles acted more strongly to reduce pigmentation at more distal positions (cyan and gray lines, Fig. 3H; for *fla^p^* ry slope = −1.05 ± 0.02 and for *SULF^p^* slope = −1.14 ± 0.003). Thus, *fla^p^* and *SULF^p^* reduce *FLA* activity in cis and trans with increasing effect distally. The *SULF^p^ r_y_* gradient sloped down to lower values than that of *fla^p^*, indicating that the *SULF^p^* trans effect exceeded the *fla^p^* cis effect. The proximodistal gradients in pseudomajus allele action, as well as the shallow proximodistal gradient of yellow in *A.m.m.* var. *striatum*, may involve responses to proximodistal prepatterns established earlier in development (*19*, *20*).

A simple gene interaction model is that for each position in the transect, the combined effect of *fla^p^* and *SULF^p^* on yellow intensity is equal to the product of the effects caused by each locus alone (i.e., the loci interact multiplicatively). Multiplying the *r_y_* values of *SULF^p^* and *fla^p^* together gave a steeper proximodistal gradient (red line, Fig. 3H), similar to the *r_y_* gradient determined experimentally from plants carrying both *SULF^p^* and *fla^p^* (dark blue line). Thus, *SULF^p^* and *fla^p^* alleles interact in a broadly multiplicative manner to steepen the yellow flower color gradient.

### Selection intensity correlates with effects of *FLA* and *SULF* alleles on the yellow gradient

**Fig. 4.**
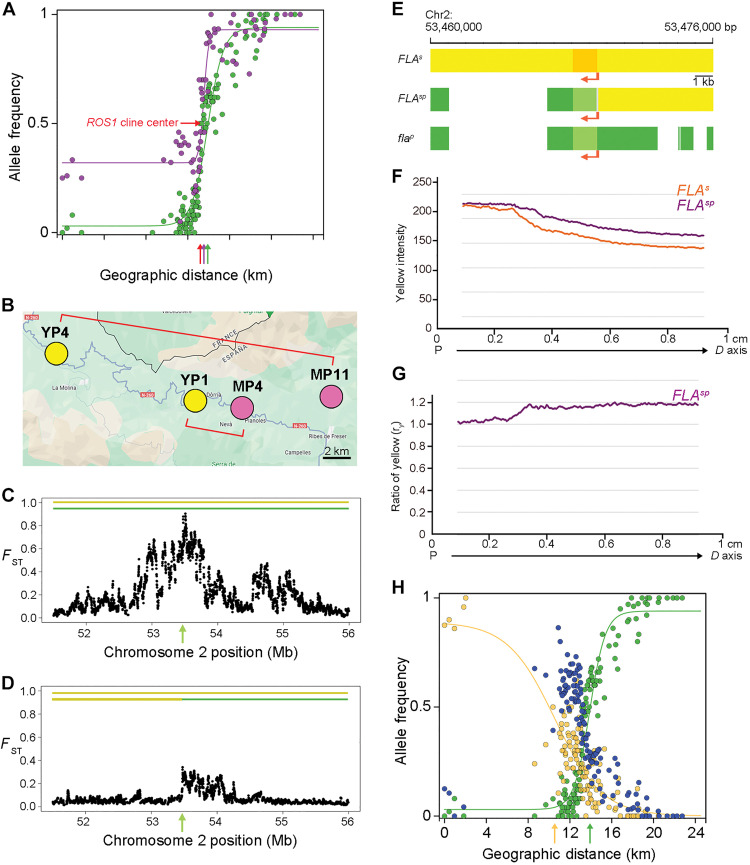
Cline and recombinant analysis. (**A**) SNP allele frequencies indicative of *SULF^p^* (purple points) and *fla^p^* (green points) clines across the hybrid zone (HZ) from *A.m.m.* var. *striatum* (west) to *A.m.m.* var. *pseudomajus* (east) flanking populations. On the basis of sigmoid fits, cline widths are about 1 km for *SULF^p^* and 3 km for *fla^p^*, with cline centers marked by arrows (including *ROS* in red). The nonzero frequency of the *SULF* pseudomajus SNP on the *A.m.m.* var. *striatum* flank is likely caused by one or more *sulf^s^* alleles carrying the pseudomajus SNP (through recombination or rearrangement). (**B**) Location of DNA pools analyzed either side of the hybrid zone. (**C**) *FLA F*_ST_ comparison with distant populations YP4 and MP11. The major alleles present in the comparison are shown by the colored lines; *FLA^s^* (yellow) and *fla^p^* (green), the *FLA* coding region by a green arrow. (**D**) *FLA F*_ST_ comparison with near populations YP1 and MP4. Lines show the major alleles; *FLA^s^* (yellow) and *FLA^sp^* (yellow-green) with the FLA coding region indicated (green arrow). (**E**) Schematic showing the genomic structures of the three different *FLA* alleles found in the HZ. *FLA^s^* (yellow), *FLA^sp^* (5′ region in yellow, breakpoint in gray, coding light green, and 3′ region in darker green), and *fla^p^* (green). Transcription direction shown by arrows. Indels shown as white gaps relative to the reference *A.m.majus*. *FLA^sp^* has the 5′ region of *FLA^s^* joined to the coding and 3′ of *fla^P^*. (**F**) YP of face region for the *FLA^s^* and *FLA^sp^* alleles. (**G**) Ratio of yellow intensity, *r_y_*, of the *FLA^sp^* allele versus *FLA^S^* allele. (**H**) *FLA^s^* (yellow), *fla^p^* (green) and *FLA^sp^* (dark blue) allele frequencies in demes across the HZ, with clines fitted for *FLA^s^* and *fla^p^*. Cline centers shown by arrows and widths are about 3 km for *fla^p^* and 9 km for *FLA^s^*.

Both *ROS/EL* and *SULF* exhibit steep clines in allele frequency centered at a similar position at a hybrid zone (*10*, *15*). Recombination between SNPs and causative mutations is expected to increase cline width, so we chose the steepest clines to estimate cline widths. On the basis of symmetrical fits (*21*), this gave estimated cline widths of about 0.8 to 1 km (95% confidence interval) for *ROS/EL* and 0.9 to 1.25 km for *SULF*. Genotyping with SNPs from the *FLA* upstream region revealed a cline at a similar geographical center though less steep than the *SULF* cline, being two to four times wider (2.6 to 3.6 km) (Fig. 4A). Cline width is approximately proportional to σ/√*s*, where σ is the dispersal distance and *s* is the selection coefficient (*9*). As dispersal distance should be similar for different loci, a two- to fourfold greater cline width suggests that the selection coefficient for *FLA* is four to 16 times less than for *SULF*. The weaker selection on *FLA* may reflect the smaller effect of *fla^p^* (i.e., higher values of *r_y_*) on yellow color compared to *SULF^p^* (Fig. 3H).

The relationship between phenotypic effect and fitness could be further investigated through analysis of a recombinant *FLA* allele found to be prevalent in the hybrid zone. Comparisons of populations further away from the hybrid zone center (populations YP4 and MP11, Fig. 4B) gave a broader and taller *F*_ST_ island around *FLA* (Fig. 4C) than comparisons between nearby populations (YP1 and MP4, Fig. 4D). SNP analysis revealed that this difference was accounted for by a recombinant allele, hereafter named *FLA^sp^*, at a high frequency near the center of the hybrid zone. *FLA^sp^* had the upstream region derived from *A.m.m.* var. *striatum* and the downstream region derived from *A.m.m.* var. *pseudomajus* (Fig. 4E). The recombination breakpoint was located between a SNP at −64 and +40 relative to ATG start codon of *FLA* (fig. S6). For distant population comparisons, the predominant allele on the *A.m.m.* var. *striatum* side was *FLA^s^*, giving a broad *F*_ST_ island, whereas in the closer populations, the predominant allele on the *A.m.m.* var. *striatum* side was *FLA^sp^*, truncating the island after the recombination breakpoint.

Analysis of petal transects showed that *FLA^sp^* gave a similar gradient in yellow to *FLA^s^* but slightly shallower (Fig. 4F). The steeper yellow gradient conferred by *fla^p^* compared to these alleles (Fig. 3G) therefore largely reflects its cis-acting upstream region (absent in *FLA^sp^* and *FLA^s^*). The *r_y_* values for *FLA^sp^* were greater than 1 and sloped upward along the proximodistal axis (slope = 0.22 ± 0.009), showing that *FLA^sp^* boosts *FLA* expression slightly relative to *FLA^s^* distally (Fig. 4G)*.* Thus, the downstream region of *FLA^s^* (absent in *FLA^sp^*) contains cis-acting elements that attenuate expression in distal petal positions.

On the basis of the above analysis, we used SNPs from various positions in the *FLA* locus to infer frequencies for three alleles: *fla^p^*, *FLA^s^*, and *FLA^sp^* (Fig. 4H). The *FLA^s^* cline (yellow) was wider than the *fla^p^* cline (8.5 to 10.1 km compared to 2.6 to 3.6 km), indicating that selection on *FLA^s^* was about six to 15 times lower than on *fla^p^*. *FLA^sp^* (dark blue) showed a peak in frequency to the left of the *fla^p^* cline center.

The high frequency of the *FLA^sp^* allele near the center of the hybrid zone most likely reflects its selective advantage in a hybrid genetic background. The hybrid zone is at least 100 years old and may be considerably older (*10*). Suppose initial contact led to the formation of complementary frequency clines for *FLA^s^* and *fla^p^*, with geographic centers coincident with the clines of magenta-controlling loci *ROS EL.* Many plants on the left side of the cline center would have yellow flowers with overlapping weak magenta caused by introgression of *ROS^p^ el^p^* alleles. In this context, a recombinant allele at *FLA* that enhanced yellow, *FLA^sp^*, may have had a selective advantage over parental *FLA^s^* by over-riding the dulling effect of magenta. *FLA^sp^* would then have been swept to high frequency in this geographical location. Outside of this location, *FLA^sp^* may have lower fitness because it confers excess yellow (on *A.m.m.* var. *striatum* side) or a gradient that is too flat (on *A.m.m.* var. *pseudomajus* side) to guide pollinators effectively.

The selective sweep of *FLA^sp^* would have led to subdivision of the hybrid zone into a sequence of three regions, each with a different predominant *FLA* allele: a left region with high *FLA^s^* frequency, a middle region with high *FLA^sp^*, and a right region with high *fla^p^*. Selection, and thus cline width, would reflect fitness differences between *FLA* alleles in adjacent regions. The large cline width for *FLA^s^* (8.5 to 10.1 km) would correspond to a small fitness difference between *FLA^s^* (left region) and *FLA^sp^* (middle region), correlating with a small phenotypic effect of *FLA^sp^* relative to *FLA^s^* (Fig. 4F). The narrower cline width for *fla^p^* (2.6 to 3.6 km) would correspond to a greater fitness difference between *FLA^sp^* (middle region) and *fla^p^* (right region), correlating with a greater phenotypic effect of *fla^p^* relative to *FLA^sp^* (Figs. 3G and 4F). The *fla^p^* cline (2.6 to 3.6 km) is still wider than the cline for *SULF^p^* (0.9 to 1.25 km), indicating weaker selection on *fla^p^*, consistent with its weaker phenotypic effect (Fig. 3H). Thus, selection coefficient broadly correlates with the strength of phenotypic effect, which likely reflects salience of visual signals to pollinators.

### Two additional loci shape the yellow gradient through multiplicative interactions

Recently, three additional loci controlling flower color differences between *A.m.m.* var. *pseudomajus* and var. *striatum* were identified by genome scans for clines (*21*) and phylogenetic signatures (*22*): one affecting magenta, *RUBIA* (*RUB*), and two affecting yellow, *AURINA* (*AUN*) and *CREMOSA* (*CRE*). These loci have weaker phenotypic effects than the *ROS EL* and *SULF* loci, and do not exhibit strong *F*_ST_ peaks. *AUN* likely encodes aureusidin synthase (AS1), which catalyzes the step before 4′CGT in the aurone synthesis pathway (*23*). *CRE* may encode an *O*-methyltransferase which could affect yellow by altering the flux of aurone precursors or by methylation of aurones. Another candidate gene for *CRE* is a *SCARECROW*-like transcription factor (*22*).

**Fig. 5.**
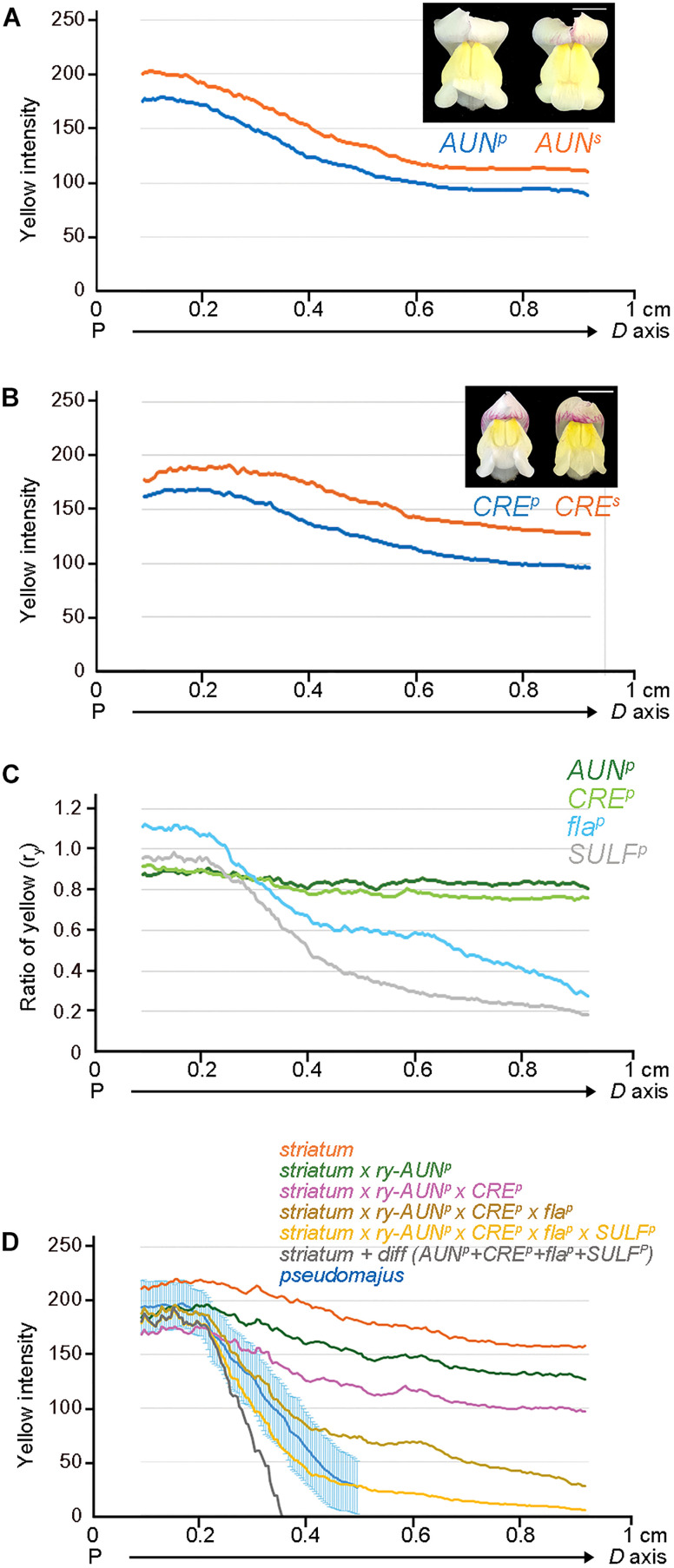
Effects of *AUN* and *CRE* and multiplicative model. (**A**) YP of face region for the *AUN^s^* and *AUN^p^* alleles in a *sulf^s^/sulf^s^ ros^s^/ros^s^ FLA^s^/FLA^s^ CRE^p^/CRE^p^* background example flowers shown. (**B**) YPs for the *CRE^s^* and *CRE^p^* alleles in a *sulf^s^/sulf^s^ ros^s^/ros^s^ FLA^s^/fla^p^ AUN^s^/AUN^p^* background. Example flowers shown. (**C**) Ratio of yellow (*r_y_*) of the *AUN^p^* versus *AUN^s^* allele, and *CRE^p^* versus *CRE^s^*, with *fla^p^* versus *FLA^s^* and *SULF^p^* versus *sulf^s^* shown for comparison. Linear regression analysis of *AUN r_y_* slope = −0.08 ± 0.005 and for *CRE* ry = −0.2 ± 0.006, where error bounds are twice the SD of the slope. (**D**) Gradient in yellow obtained by multiplying the *A.m.m.* var. *striatum* profile (orange) by *r_y_* values of different loci in combination. *A.m.m.* var. *pseudomajus* profile shown in blue, with twice the SE shown as vertical blue bars (data points only go up to 0.5 cm, as distal magenta interferes with measurement of yellow). Predicted multiplicative interaction for all loci shown in yellow, additive prediction in gray.

Yellow profiles (YPs) of *AUN* and *CRE* in genetic backgrounds fixed at the other loci showed that pseudomajus alleles reduce yellow in both cases (Fig. 5, A and B, and figs. S7 and S8). Values of *r_y_* gave a slight negative slope, significantly different from zero, indicating that pseudomajus alleles steepened the gradient in yellow but to a lesser extent than *FLA* or *SULF* (Fig. 5C), at least in the genetic backgrounds analyzed.

To determine how the four yellow loci, *AUN*, *CRE*, *FLA*, and *SULF* interact, we first assumed a multiplicative model (i.e., multiplying their values of *r_y_* together) and calculated the yellow intensity they would confer by progressively introducing more pseudomajus alleles into *A.m.m.* var. *striatum* (Fig. 5D). Introduction of pseudomajus alleles progressively steepened the gradient of yellow. The yellow intensity in the most distal position shown (0.92 cm) was reduced by 97% when all four were combined. The predicted gradient of yellow when all four alleles combined was within the error bounds of that observed for the *A.m.m.* var. *pseudomajus* flowers (only distances up to 0.5 cm are shown, as magenta interferes with yellow pigment estimates beyond this). An additive model gave a good fit for distances up to 0.3 cm but deviated significantly beyond this. Thus, a multiplicative model gave a better overall fit.

The steep gradient in yellow color in *A.m.m.* var. *pseudomajus* is therefore established through multiplicative interactions between four loci. The effect of single alleles at some loci (e.g., *AUN* and *CRE*) is hardly perceptible to the human eye yet may be salient enough to pollinators to influence their behavior. Collectively, their effects multiply to produce a steep gradient. Our findings are consistent with genome-wide surveys in yeast, which have shown that functional gene interactions are most commonly multiplicative (*24*).

For the observed clines in allele frequency to be maintained, selection must prevent alleles at each locus from introgressing from one subspecies into the other. There are no obvious differences in physical environment or pollinators between *A.m.m.* var. *striatum* and *A.m.m.* var. *pseudomajus* populations either side of the hybrid zone (*25*, *26*). Selection therefore most likely maintains the clines because of the epistatic interactions between loci controlling magenta (*ROS*, *EL*, and *RUB*) and yellow (*SULF*, *FLA*, *AUN*, and *CRE*) flower color. In *A.m.m.* var. *pseudomajus*, the seven loci interact to produce a flower with a yellow highlight against a largely nonoverlapping a magenta background. An allele that flattens the yellow gradient, causing it to overlap with magenta would muddy the colors making the flower less attractive to pollinators. In *A.m.m.* var. *striatum*, the seven loci interact to produce a flower with a magenta highlight on a graded yellow background. An allele that further steepens or flattens the yellow gradient in this context may reduce pollinator attractiveness or guidance. In a genetic background where alleles at loci have been mixed, as in the center of the hybrid zone, alleles such as the recombinant, *FLA^sp^*, can be favored over parental ones.

Our results indicate that honing of the yellow developmental gradient has been shaped with great precision through selection acting on multiple loci rather than through modifications at a single locus, consistent with the notion of evolutionary tinkering (*27*). The phenotypic effect of individual alleles can be very small yet confer fitness differences sufficient for selection to maintain steep clines between hybridizing populations. Fitness differences depend on epistatic interactions with other loci, notably those controlling magenta color. Thus, depending on genetic context, different gradients may be favored.

Precise shaping of developmental gradients has been described for other systems, such as the Bicoid morphogen gradient in the *Drosophila* egg (*4*). Unlike the yellow gradient in *Antirrhinum*, the Bicoid gradient is established through diffusion and dynamic control of morphogen degradation, rather than in response to a prepattern. It is not known how selection has tuned the Bicoid gradient but it may have involved alleles that slightly shift, steepen, or flatten the gradient by modifying production, diffusion, or degradation parameters, which may vary spatially across the embryo (*28*). Some of these alleles may have been at Bicoid, but others may have been at different loci controlling these parameters. Thus, selection on multiple loci, each with alleles having modest effects, may provide a general mechanism for evolutionary shaping of developmental gradients.

## MATERIALS AND METHODS

### Plant materials

The locations of the *Antirrhinum* species accessions used in this study were mapped (fig. S9). An interactive location resource is found at: http://antspec.org/.

Seed from Accessions and John Innes stocks were grown on soil-compost mixes in pots or trays, outside on benches in the summer, or in the greenhouse with supplemental light in winter as described (*29*). Lines from self-incompatible species were maintained by intersibling crossing.

*A. majus majus* (*FLA^m^/FLA^m^; SULF^m^/SULF^m^; ROS^m^ el^m^/ROS^m^ el^m^*) John Innes reference line JI7 was used to make the Snapdragon Genome Reference Sequence (http://bioinfo.sibs.ac.cn/Am/) and used in crosses for studying *FLA^m^* alleles. *A.m.m.* var. *pseudomajus* (*fla^p^/fla^p^; SULF^p^/SULF^p^; ROS^p^ el^p^/ROS^p^ el^p^*) reference line seeds were collected from allopatric populations near Ventola ~5 km east of the Pyrenees hybrid zone.

*A.m.m.* var. *striatum* (*FLA^s^/FLA^s^*; *sulf^s^/sulf^s^*; *ros^s^ EL^s^/ros^s^ EL^s^*) reference line seeds were collected from allopatric populations near La Molina ~10 km west of the Pyrenees hybrid zone. Self-incompatible wild *Antirrhinum* lines were maintained by intercrossing siblings at each generation.

Wild Accession pools of *A.m.m.* var. *pseudomajus* or *A.m.m.* var. *striatum* were harvested from ~30 to 50 individuals from different locations as described (*9*, *18*, *19*).*A.m.m.* var. *pseudomajus* and *A.m.m.* var. *striatum* accessions used were also maintained from wild collected seed by intercrossing siblings and their original locations mapped (fig. S9).

### F2s and introgressions

#### A.m.m. var. pseudomajus x A.m.m. var. striatum F2

*A.m.m.* var. *pseudomajus* from Ventola plant J1428 seed capsule 2 was sown to give individual V163-36. *A.m.m.* var. *striatum* from La Molina plant J1324 seed capsule 5, was sown to give individual V206-40. V163-36 was crossed with V206-40 to generate F1 plants (Y132-1 to 5) which were self-incompatible and therefore sibs were intercrossed to give the F2 family J109.

#### A.m.m. var. striatum x A.m. majus

*A.m.m.* var. *striatum* from La Molina plant J1160 seed capsule 3 was sown to give individual V189-16. *A.m. majus* JI7 was crossed with V189–16 to generate F1 plants (Y137) that were self-pollinated to give F2 family J106.

#### A.m.m. var. pseudomajus x A.m. majus

*A.m.m.* var. *pseudomajus* came from Ventola plant J428 seed capsule 2, which was sown to give individual V163-36. *A.m. majus* JI7 was crossed with V163-36 to generate F1 plants (Y135) that were self-pollinated to give F2 family J108.

### F3s and F4s

For Fig. 2C, an F4 *sulf^s^/sulf^s^* population was generated from intercrossing individuals of the *A.m.m.* var. *pseudomajus* x *A.m.m.* var. *striatum* F2 family J109 above to give F3 families K115 and K116. Individuals from these families were crossed to give F4 family L116 (*ros^s^ EL^s^/ros^s^ EL^s^; sulf^s^/sulf^s^*) segregating for *FLA^s^* and *fla^p^*.

For Fig. 2D, an F4 *SULF^p^/SULF^p^* population was generated from intercrossing individuals of the *A.m.m.* var. *pseudomajus* x *A.m.m.* var. *striatum* F2 family J109 above to give F3 family K113. Two sibs from this family were intercrossed to give F4 family V162 (*SULF^p^/SULF^p^*) segregating for *ROS^p^ el^p^* and *ros^s^ EL*^*s*,^ and for *FLA^s^* and *fla^p^*. For ease of scoring, only *ros^s^ EL^s^/ros^s^ EL^s^* individuals were analyzed.

To obtain all four combinations of *SULF^p^* versus *sulf^s^* and *FLA^s^* versus *fla^p^* for analysis in Fig. 3, we used *ros^s^ EL^s^/ros^s^ EL^s^* individuals families L116 and V162 families above. They were further genotyped and selected as fixed for *CRE^s^/CRE^p^* and *AUN^s^/AUN^p^*, but segregating for *FLA^s^* and *fla^p^*, before selecting 10 individuals for analysis.

The effects of other loci analyzed in Fig. 5 used the populations L116 for *CRE* and V164 for *AUN*. Genotyping of family L116 identified *sulf^s^/sulf^s^; ros^s^ EL^s^/ros^s^ EL^s^; FLA^s^/FLA^s^*; *CRE^s^/CRE^s^* individuals segregating for *AUN^s^* and *AUN^s^*. Family V164 provided individuals with genotype *sulf^s^/sulf^s^; ros^s^ EL^s^/ros^s^ EL^s^; FLA^s^/fla^p^; AUN^s^/AUN^p^* segregating for *CRE^s^* and *CRE^p^*. Again, 10 individuals with the different *CRE* genotypes were color profile analyzed.

### Leaf genomic DNA isolation

For genotyping, three to six small young leaves (~1 cm long; total 100 to 200 mg) were collected in Eppendorf tubes and frozen in liquid nitrogen. Large numbers were collected in 96 tube or plate format and frozen at −20°C before isolation by the QIAGEN DNeasy 96 Plant Kit or an in-house Genotyping Service method as described in: Wheat and Barley DNA Extraction in 96-well Plates – MASWheat (yumpu.com).

For genomic DNA, samples from the greenhouse were stored at −80°C until extraction. Leaf samples collected from field locations in France and Spain were either placed in bags in silica, or stored in moist paper towel and kept cool at 4°C until they could be Courier Posted by overnight delivery to the lab in the UK and frozen at −80°C on arrival. Genomic DNA was isolated by a CTAB method using ~2 to 10 g of leaves harvested either from a single individual or as pooled samples as described (*30*).

### Photography

Flowers were placed on black velvet with a scale bar and Small Grey & Colour Separation Chart (Danes Picta BST13) for color, light level, and white balance monitoring. We used an Olympus XZ-1 (10 Megapixels) camera with side/overhead lighting via table lamps fitted with halogen 42 W 630 lumen (2800 k) warm white light bulbs. Camera settings were set to the closest White Balance of 3000 K, no flash, Macro On, F stop 8.0, exposure time 1/20 to 1/40 s, ISO 200, RAW images, aspect 4:3, and high definition.

### Phenotyping

Yellow and magenta patterns were scored for flowers taken fresh from each individual plant. Scores were made by eye using the numerical system as described (*13*). Photographs of each flower, face, and side views were taken for reference and re-checking. Flowers were also cut in half to give lower ventral/lateral petals separated from upper/dorsal. These allowed optimal imaging of yellow color in the face region as used in ImageJ.

### ImageJ analysis of flower color profiles

Ten different individual flower photographs were selected of the lower half of each flower and foci to the edge of petal lobes. Each original jpg was first adjusted in Photoshop using image-adjustments-levels to standardize the white balance, using the white reference color chart square included in all photographs. Images were collected in one Photoshop image at 600 dpi RGB and all backgrounds made black.

Each individual was adjusted to the same size of 1 cm from foci to the most distal point on the petal face. Flowers were then orientated so that this foci to distal point transect was vertical. The image was saved as a jpg and opened in ImageJ.

In ImageJ a 1 pixel width line was drawn to run through the vertical foci to the edge of the petal lobe. The flower image was split into the three-color channels and the blue, green, and red profiles collected in Excel. We calculated yellow as green pixels minus blue pixels. Fractional effects were calculated from the YP at each point along the transect of the petal face: ratio of yellow *r_y_* = YP of pseudomajus value/YP of striatum value. This allowed us to calculate the effect of any pseudomajus allele on any striatum YP. For example, the *r_y_* for each locus, *FLA*, *SULF*, *AUN*, and *CRE* could be used against the YP of an *A.m.m.* var. *striatum* to see how close it makes the profile to an *A.m.m.* var. *pseudomaju*s profile.

Calculation for ADDITIVE MODEL was as follows: yellow intensity profile for *AUN^p^* = *striatum* value at any point × [1 − (1 − ry *AUN^p^*)] and yellow intensity profile for *AUN^p^* + *CRE^p^* = *striatum* value at any point × [1 − (1 − ry *AUN^p^*) + (1 − ry *CRE^p^*)] and similarly for *n* genes. Calculation for MULTIPLICATIVE MODEL was as follows: yellow intensity profile for *AUN^p^* = *striatum* value at any point × ry *AUN^p^* and yellow intensity profile for *AUN^p^* × *CRE^p^* = *striatum* value at any point × ry *AUN^p^* × ry *CRE^p^*, and similarly for *n* genes.

### *F*_ST_ analysis

The comparison of population pools for *F*_ST_ determination was as described in (*10*).

### Measuring Chr2 recombination rate

Families were generated (see the “Plant materials” section above) that allowed recombination between *A.m.m.* var. *pseudomajus* and *A.m.m.* var. *striatum* (J109), *A.m.m.* var. *pseudomajus* and *A.m. majus* (J108), or *A.m.m.* var. *striatum* and *A.m. majus* (J106). Each F2 family was genotyped with KASP markers (table S2) along the chromosome. The resulting genotypes and recombination events were used to calculate recombination frequencies along chr2, based on the number of gametes screened to give fig. S2.

### Genotyping plants

The KASP Genotyping method (LGC) was as described (*22*), and the fluorescence signals discriminating homozygous or heterozygous alleles were detected by a BioRad CFX96 Lightcycler and the data processed using the BioRad CFX Manager software version 3.1.

The KASP method (LGC Genomics) was used to identify *FLA*, *SULF*, *AUN*, and *CRE* alleles using the oligo sets listed in table S1 below. AFLP markers were also used to genotype the *EL* locus and confirm *CRE* families (table S1). Standard polymerase chain reaction (PCR) conditions were used, with annealing at 55°C and extension steps at 72°C of 1 min. PCR products were run on 1% (w/v) agarose gels, stained with ethidium bromide (~0.5 μg/ml) and photographed under ultraviolet light.

### Flower color ranking

Flower photographs included the Danes Picta BST13 color chart, allowing all flower images to be white-balanced equivalently in Photoshop. By eye, each image was assessed for the yellow spread/intensity and positioned within a ranking order. Ranking order was continually updated as more flowers were analyzed. The final ranking was divided into four quartiles. Within each quartile set, the *FLA* genotypes were determined for each individual, and the numbers of *A.m.m.* var. *pseudomajus (p)* and *A.m.m.* var. *striatum (s)* alleles were recorded.

### RNA-seq

Total RNA was isolated from petal tissues using the Qiagen RNeasy Plant Mini Kit, including DNaseI treatment. For a comparison of *A.m.m.* var. *pseudomajus* (accession Ac1266) and *A.m.m.* var. *striatum* (accession Ac1125), we harvested petal lobes from dissected flowers just before opening. Three independent samples were used for reproducibility, and each sample was a pool of three individuals, each contributing one to two flowers. Samples were sent to the Earlham Institute, Norwich, UK for in-house library and Illumina sequencing runs to give 2 × 75 bp paired-end sequencing of strand-specific libraries.

For a comparison of *sulf^s^/sulf^s^ FLA^s^/FLA^s^* with *sulf^s^/sulf^s^ fla^p^/fla^p^* (in the segregating F2 population J109), only upper/dorsal petal lobes were used. As above, triplicate samples were analyzed as described above. These samples were sent to Novogene, Cambridge, UK and processed as above. All RNA-seq data were processed, mapped, and analyzed as described (*19*).

### RNA in situ

*FLA* probes and method were as described (*31*). In addition, pre–in situ photographs were taken of the wax sections as the flower pigments had been preserved and thus highlighted the patterns of yellow, magenta, and nonpigmented regions. Pigmentation was subsequently lost on dewaxing and processing of tissue for in situ.

### Geographic cline analysis

#### Plant sampling and SNP genotyping

At the Planoles hybrid zone, Los Pironeos, Spain, individual plants were sampled for leaf material and flowers (color phenotyping) and geolocated (with Trimble GEOX) as part of another study building a pedigree at the site over generations. These samples came from surveys over a period of 6 to 8 weeks each year (late May/early June to late July) between 2009 and 2023, where teams repeatedly searched along two valley roads and in the surrounding vegetation and sampled all flowering *Antirrhinum* plants found. The same location was visited four to six times each year (~every 10 to 12 days), ensuring we captured plants that started flowering at different times during the season. DNA was extracted from dried leaf material and SNP genotyped using the KASP platform (*32*) at each of six color loci including *ROSEA* (ros_assembly_543443), *ELUTA* [ref. (*9*)], *SULFUREA* (s91_39699) and *FLAVIA* (s316_93292 and s316_257789), as well as the other divergent loci in recently described color genes (*RUBIA* – s261_720757 and *CREMOSA* – s1187_290152). The *SULFUREA* marker was chosen as that showing the steepest cline. The frequency of the *SULF^p^* allele did not approach zero on the *striatum* (left) flank most likely because of recombinant alleles (genotyping revealed that 24% of plants judged to be homozygous *sulf^s^* based on phenotype carried the *SULF^p^* marker). The loci were genotyped for a total of >12,000 individuals across 15 years for a larger pedigree study along the hybrid zone and spanning the transition in color phenotypes from yellow *A.m.m.* var. *striatum* to magenta *A.m.m.* var. *pseudomajus*.

### Cline fitting with simulated annealing

The hybrid zone at Planoles lends itself to one-dimensional cline fitting due to the narrowness of the habitat along a primarily east–west valley with high mountains to the north and south that are above the altitude limit of these species. Here, *Antirrhinum* plants are primarily found within 100 m either side of two roughly parallel roads that run up the valley. First, allele frequencies and genotype counts were determined within a 200 × 200 m grid of discrete demes. This scale minimized deficits from Hardy-Weinberg equilibrium (see Results) while ensuring sufficient sample size within demes (mean = 40, SD = 20, range of 10 to 200) and yielded 125 demes across the hybrid zone. Only one marker within *ROS1* displayed significant departures from HW with a deficit of heterozygotes (*F* > 0) even at small spatial scales (<50 m). Therefore, we expect, based on theory, that this feature may generate narrower cline widths than expected.

The clines were characterized for each color locus, using a modified version of a custom R script (slowClines) as described (*9*, *22*). This script uses fits a symmetric sigmoid cline with five parameters

$$\hat{p}=\frac{p_{0}+\left(p_{1}-p_{0}\right)}{1+\text{exp}\left[-4\left(\frac{x-c}{w}\right)\right]}$$

where c = cline center, w = cline width (1/gradient), $p_{0}$ = allele frequency at the asymptote in the west (*A.m.m.* var. *striatum* parental allele frequency), and $p_{1}$ = allele frequency at the asymptote in the east (*A.m.m.* var. *pseudomajus* parental allele frequency) and $F_{\text{ST}}=\text{var}\left(p\right)/\bar{p}\left(1-\bar{p}\right)$. For the $F_{\text{ST}}$ parameter, we fitted a β-binomial error term to account for the variance in allele frequencies across demes and to control for population structure along the cline. Before commencing cline fitting algorithm, the larger dataset was randomly thinned within each deme to reduce the computational time, resulting in 2900 to 3300 individual genotypes across ~100 demes.

We use a metropolis-hastings (simulated annealing) algorithm to sample the likelihood surface of the cline fit. We begin the algorithm with a random set of parameters, which are changed randomly and the log likelihood (log*L*) is computed at each iteration. When the next iteration log*L*″ has a greater log*L* than the previous likelihood log*L*′ (i.e., log*L*″ > log*L*′), the new parameters are accepted. If the next iteration is lower (log*L*″ < log*L*′), we accept with a probability log*L*″/log*L*′. To ensure ample exploration of the likelihood surface, the jump size for the next set of parameters are adjusted by a factor of 1.05 when accepted (accept scale) and by (1/1.05) when parameters are rejected (reject scale). After some tests of different accept and rejection scales, we found these values achieved efficient mixing and exploration of the likelihood surface with an acceptance rate ~0.5. This algorithm was run for 50,000 iterations with a burn-in = 2000. From this, we find the most likely cline parameters, maximum logL and assume the likelihood surface follows a chi-square distribution to find −2 logL max 95% credible regions. We visually inspected the joint likelihood surface for each run. Each run was repeated with randomly chosen starting parameters to ensure reproducibility.

Before cline fitting, the allele frequencies in demes were collapsed to one dimension, by using a linear transect through the approximate cline center (*p* = 0.5 isocline of *ROS1*). To search for the optimal transect gradient we compared cline width and maximum log*L* values with a range of gradients and intercepts centered on the *p* = 0.5 isocline through the valley (see fig. S10 for example) at each of the loci.

Comparing the optimal transect across loci, there was no common transect direction (gradient) with a best fit across all loci (fig. S11). We found that a gradient of −0.345 and intercept at 6.9 km generated the best compromise with highest likelihood (log*L*; max*LL*) or within 2 log*L* of the maximum (max*LL*) at two of the three loci (*ROS1* and *FLAVIA up*). The third locus (*FLAVIA down*) was a considerably poorer cline fit at this transect; however, comparing its best fitting transect cline parameters to the common well-fitting transect (at *ROS1* and *FLAVIA up*) showed similar centers and widths regardless of the transect chosen. Therefore, we report cline parameters for the gradient of −0.345 and intercept at 6.9 km.

### Recombinant haplotype frequencies across the hybrid zone

At the *FLAVIA* locus, we used SNP genotypes flanking either side of the promoter region to distinguish parental haplotypes from the recombinant haplotype. We denote the purebred *A.m.m.* var. *striatum* haplotype as “*FLA^s^*” and the diploid genotype as *FLA^s^/FLA^s^* the *A.m.m.* var. *pseudomajus* as “*fla^p^*” and genotype as *fla^p^/fla^p^*. Recombinant haplotypes are denoted as “*FLA^sp^*”. We use two diagnostic SNP markers (s316_93292 and s316_257789), setting genotype scores to reflect the number of copies of the *A.m.m.* var. *pseudomajus* allele, making the three possible genotypes as (i) homozygote genotypes for *A.m.m.* var. *striatum* as 0, (ii) the *A.m.m.* var. *pseudomajus* as 2 and (ii) heterozygotes as 1. Combinations of genotypes at the two diagnostic loci then allowed scoring of parental haplotypes at *FLAVIA* with the following genotype at s316_93292 and s316_257789 as:

1) *FLA^s^/FLA^s^* = double *FLA^s^* (parental *A.m.m.* var. *striatum*) = [0,0,]

2) *FLA^s^/fla^p^* = heterozygote [1,1]

3) *fla^p^/fla^p^* = double *fla^p^* (parental *A.m.m.* var. *pseudomajus*) = [2,2]

4) *FLA^sp^/FLA^s^* = *FLA^sp^* recombinant on *A.m.m.* var. *striatum* background = [0,1]

5) *FLA^sp^/fla^p^* = *FLA^sp^* recombinant on *A.m.m.* var. *pseudomajus* background = [1,2]

6) *FLA^sp^/FLA^sp^* = *FLA^sp^* heterozygous recombinant = [1,1]

This scoring was performed in a custom R script for 2700 individuals and frequencies of haplotypes calculated across the same geographic cline transect as described for diploid genotypes (see above).

Geographic cline property estimates from symmetrical sigmoid cline fitting five-parameter model for six biallelic SNP loci are given in fig. S3.

### *FLA* recombinant allele characterization

To analyze the *FLA* genomic structure and its allelic variants, we selected ~7 to 8 kb either side of the single exon of ~1.5 kb at the interval Chr2: 53,460,000 to 53,476,000. We made whole-genome sequencing of an *A.m.m.* var. *striatum* accession (Ac1256 MON, Coen ID D125), a *FLA* recombinant line originally from the Hybrid Zone (Coen ID D292) and an *A.m.m.* var. *pseudomajus* accession (Ac1097 FLO Coen ID D144). The Illumina reads from these lines were mapped against the *A.m. majus* reference genome v4, compared and indels determined by scanning in IGV (Integrative Genomics Viewer). Regions of homology were detected by BLAST at National Center for Biotechnology Information or local BLAST to the reference genome.

To define more precisely the recombination site of the Hybrid Zone *FLA* recombinant allele, revealed by its genomic structure, we used two methods. First, we used principal components analysis results to define a high confidence set of pure homozygotes (*n* = 6 for *pseudomajus* [accessions with Coen IDs D169, D180, D181, D182, D183, and D144]) and *n* = 7 for *striatum* [accessions with Coen IDs D125, D186, D187, D151, D153, D155, and D157], with a recombinant line established from the hybrid zone with Coen ID D292, for comparison. We called genotypes at biallelic SNVs across the interval 53.275 to 53.80 Mb. Individual samples were required to have a minimum depth of 5 and genotype quality of 15. The resulting 358 SNVs were filtered to select those that were diagnostically different between the two “pure” groups (*n* = 41). An example screenshot of the resulting spreadsheet is shown in fig. S12. The two SNPs defining this breakpoint were mapped to *A.m. majus* genome v3 at Chr2: 53277698 and Chr2: 53278320, an interval of 622 bp.

On viewing the region in IGV, we could further refine the interval by identifying an additional diagnostic SNV that had not fit previous filtering criteria. This site had been called as multiallelic since D169 (non-HZ *pseudomajus*) carries the striatum allele here but in phase with additional SNPs that are not seen in *striatum*. This result validated the use of this site to redefine the bounds for v3 as Chr2: 53277698-Chr2: 53277801.

Second, to confirm the above recombination region by Sanger sequencing, we amplified ~2.15 kb around the recombination site of various lines using oligos do.259 and do.460 (table S1). The lines were: *A.m.m.* var. *pseudomajus* pool MP11, *A.m.m.* var. *pseudomajus* accessions Ac1110 (CAP: Coen ID D169) and Ac1097 (FLO: Coen ID D144), *A.m.m.* var. *striatum* pool YP4, *A.m.m.* var. *striatum* accession Ac1256 (MON: Coen ID D125), and allopatric line J1152 (Coen ID D186).
