# Supplementary material for: Shaping of developmental gradients through selection on multiple loci in Antirrhinum
Source: Sci Adv. 2026 Jul 17;12(29):eadx2011. doi: 10.1126/sciadv.adx2011 (PMC13378536; doi:10.1126/sciadv.adx2011)
Supplement: Supplementary file 1 — Figs. S1 to S12 Tables S1 to S3 [file sciadv.adx2011_sm.pdf]

Science Advances
AAAS

Supplementary Materials for

Shaping of developmental gradients through selection on multiple loci in *Antirrhinum*

Desmond Bradley *et al.*

Corresponding author: Yongbiao Xue, ybxue@genetics.ac.cn; David Field, david.field@mq.edu.au; Enrico Coen, enrico.coen@jic.ac.uk

Sci. Adv. **12**, eadx2011 (2026)
DOI: 10.1126/sciadv.adx2011

This PDF file includes:

Figs. S1 to S12
Tables S1 to S3

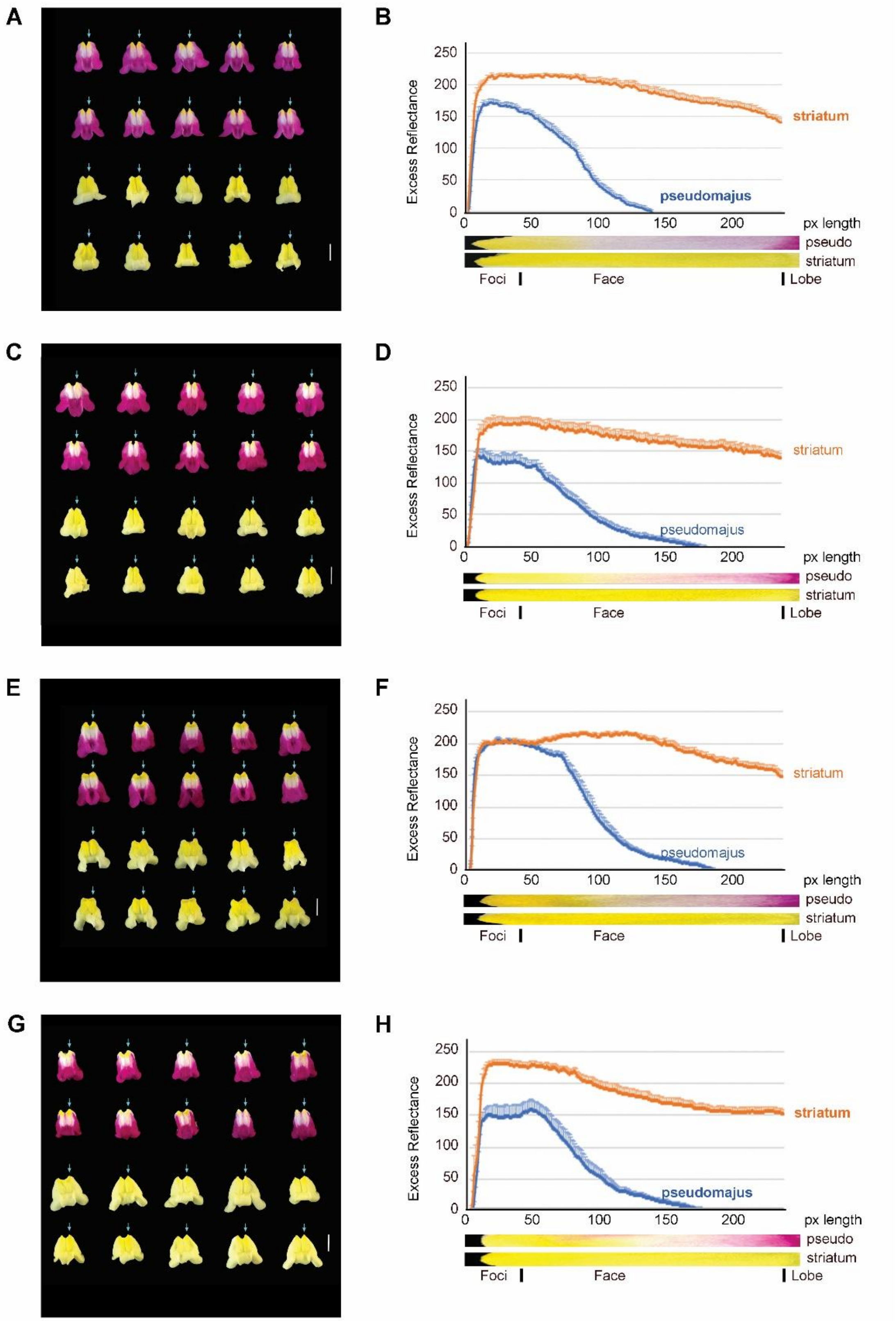
A
B
Excess Reflectance
250
200
150
100
50
0
0
50
100
150
200
px length
striatum
pseudomajus
pseudo
striatum
Foci
Face
Lobe
C
D
E
F
G
H

Fig. S1. Colour Profiles of different *A.m.m.* var. *pseudomajus* and *A.m.m.* var. *striatum* populations.

(A) Seed were collected from wild accessions of *A.m.m.* var. *pseudomajus* (location NDM) and *A.m.m.* var. *striatum* (location VCO) (see location map Supplemental fig.S9). Seed were grown in the greenhouse and, for each accession, two sibs were intercrossed and the progeny sown in 2020. From each population 10 flowers were dissected, photographed and scaled to 1 cm length for foci to the end of lobe (240 pixels in ImageJ). **(B)** The colour profiles of all 10 for each line were determined using the blue arrow as a guide for foci to end of face. Yellow = green reflectance minus blue. Their averages with SE were plotted against distance in pixels from the foci as described (Materials and Methods).
(C, D) Analysis as A,B, but using *A.m.m.* var. *pseudomajus* Accession Z-NDM-4 and *A.m.m.* var. *striatum* Z-VCO-6, from different sib intercrosses to those in A,B, and grown in 2023.
(E, F) *A.m.m.* var. *pseudomajus* and *A.m.m.* var. *striatum* seed were collected from allopatric populations either side of the Hybrid Zone in the Pyrenees and sown in the greenhouse. Exemplar (archetypal) individuals were selected and sib-intercross seed was generated. After 3 more generations, populations were grown in 2020 and analysed as described in A,B.
(G, H) *A.m.m.* var. *pseudomajus* and *A.m.m.* var. *striatum* from the same exemplar parent as E and F, but used different sib crossings and were sown in 2023, with analysis as described in A,B.

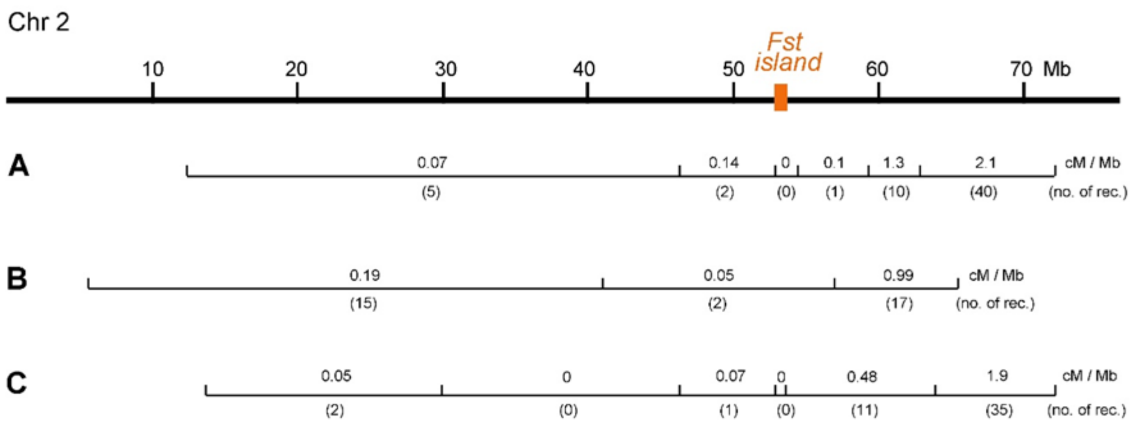

Fig. S2. Recombination analysis of chromosome 2.

A black horizontal line represents the *Antirrhinum majus majus* reference genome v3 chromosome 2. It is ~76.7 Megabases (Mb) long with an F_{ST} island of ~0.8 Mb (brown rectangle), identified from comparisons between *A.m.m.* var. *pseudomajus* and *A.m.m.* var. *striatum* population genomes. (**A**) From an F2 of *A.m.m.* var. *pseudomajus* x *A.m.m.* var. *striatum* (n=105) we used 10 markers along chr2 to find the number of recombinations for each interval (no. of rec.) to estimate the recombination frequency in centiMorgans/Mb. (**B**) The same analysis was made for an F2 of *A.m.m.* var. *striatum* x *A.m. majus* (n=112). (**C**) The same analysis was made for an F2 of *A.m.m.* var. *pseudomajus* x *A.m. majus* (n=112).

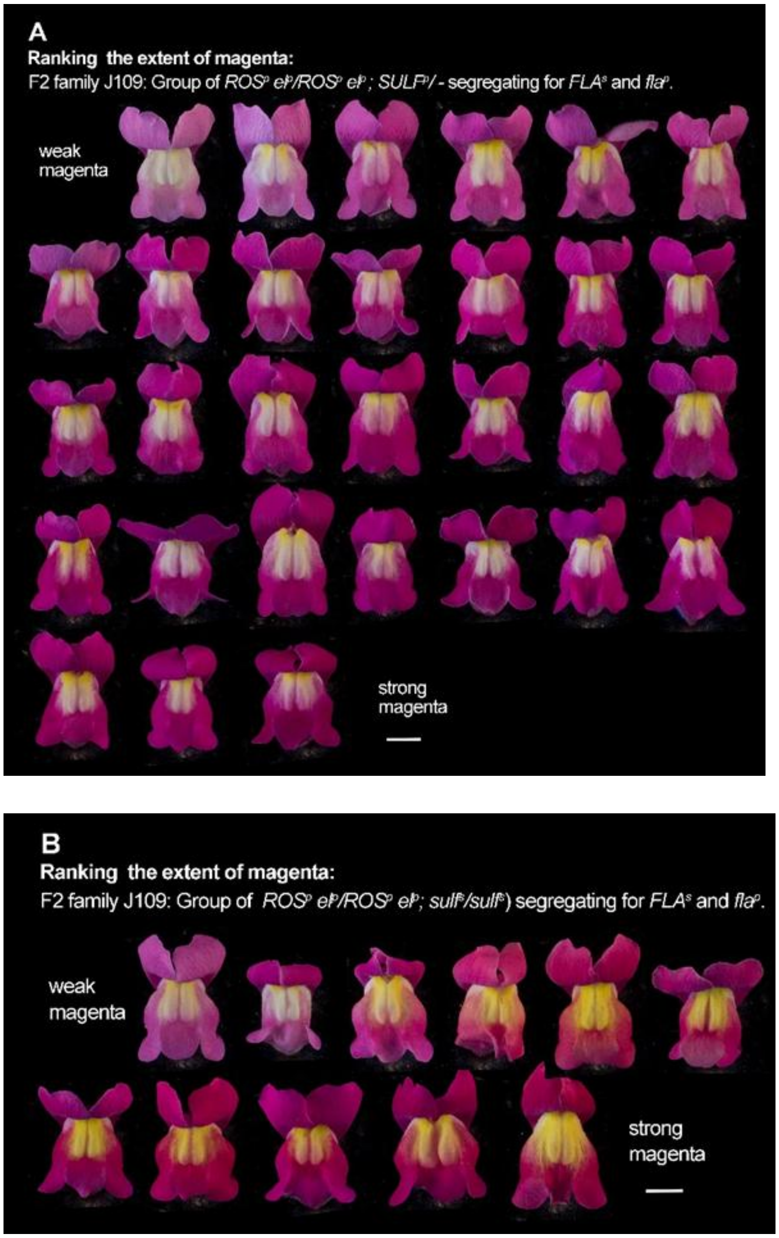
A
Ranking the extent of magenta:
F2 family J109: Group of ROS^p el^p/ROS^p el^p ; SULF^p/ - segregating for FLA^s and fla^p.
weak magenta
strong magenta
B
Ranking the extent of magenta:
F2 family J109: Group of ROS^p el^p/ROS^p el^p; sulf^s/sulf^s) segregating for FLA^s and fla^p.
weak magenta
strong magenta

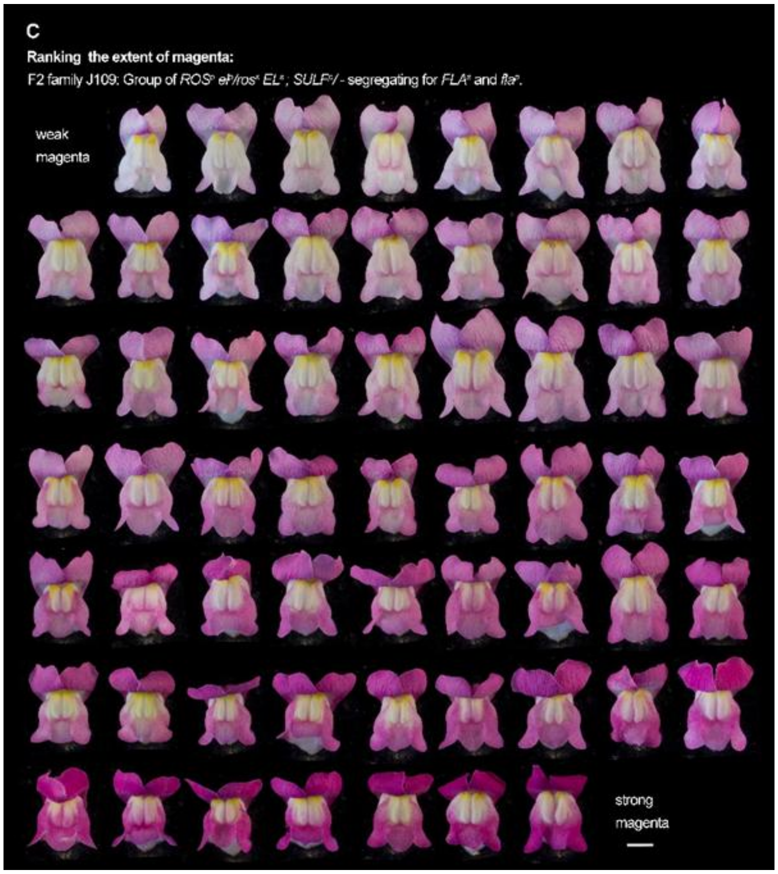
C
Ranking the extent of magenta:
F2 family J109: Group of ROS^{p} el^{p}/ros^{s} EL^{s} ; $SULF^{p}$/ - segregating for FLA^{s} and fla^{p}.
weak magenta
strong magenta

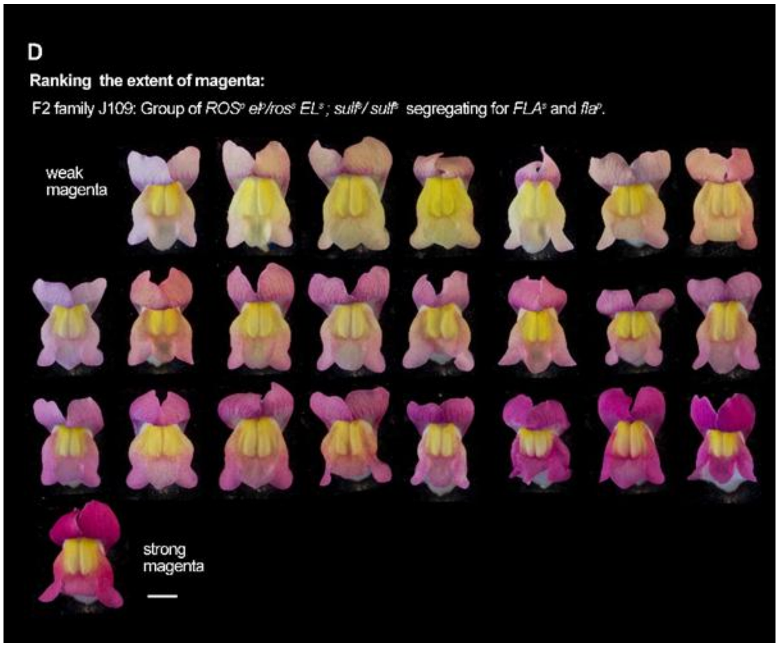
D
Ranking the extent of magenta:
F2 family J109: Group of ROS^{p} el^{p}/ros^{s} EL^{s} ; $sulf^{s}$/ $sulf^{s}$ segregating for FLA^{s} and fla^{p}.
weak magenta
strong magenta

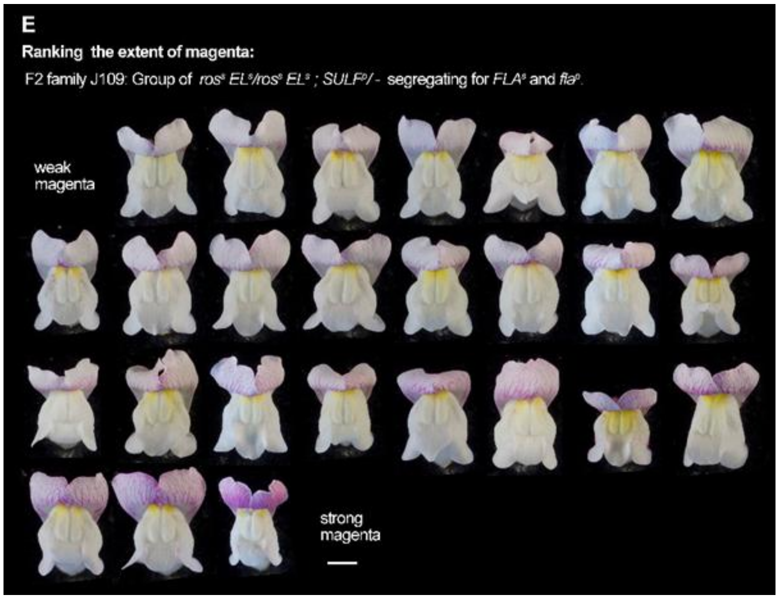
E
Ranking the extent of magenta:
F2 family J109: Group of $ros^s\ EL^s/ros^s\ EL^s$; $SULF^p/-$ segregating for FLA^s and fla^p.
weak magenta
strong magenta

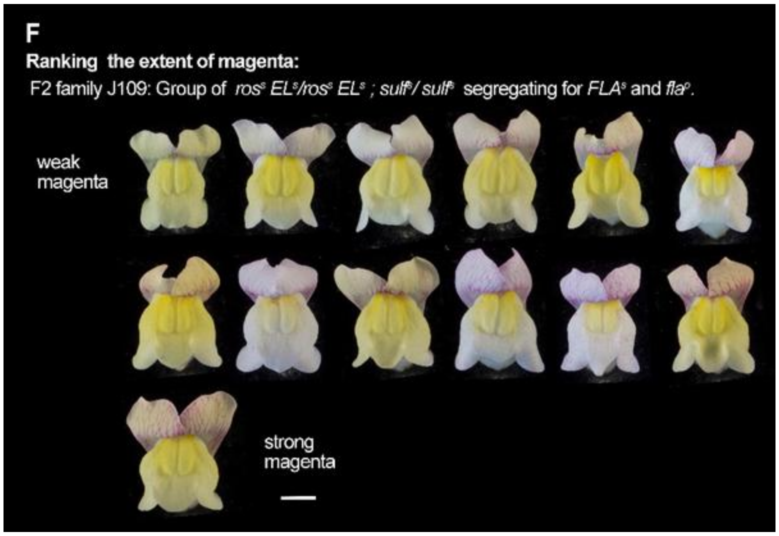
F
Ranking the extent of magenta:
F2 family J109: Group of $ros^s\ EL^s/ros^s\ EL^s$; $sulf^s/sulf^s$ segregating for FLA^s and fla^p.
weak magenta
strong magenta

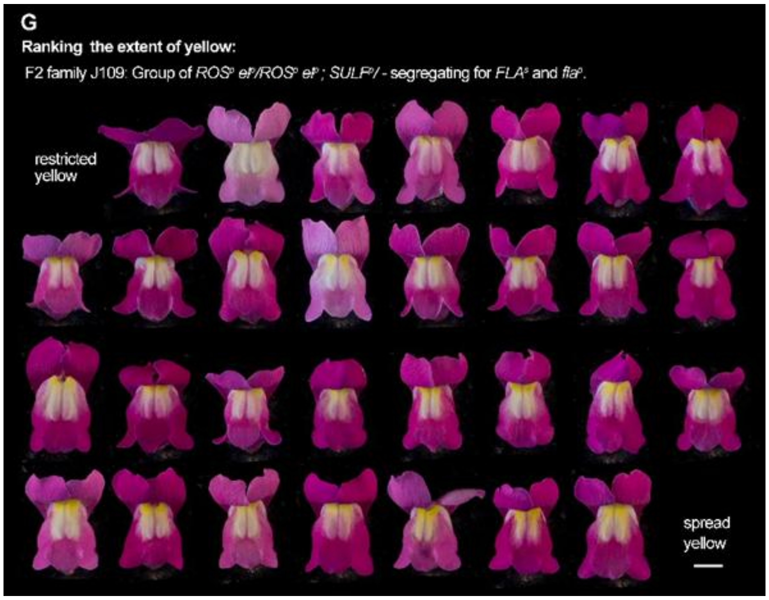
G
Ranking the extent of yellow:
F2 family J109: Group of *ROS*^{p} *el*^{p}/*ROS*^{p} *el*^{p} ; *SULF*^{p}/ - segregating for *FLA*^{s} and *fla*^{p}.
restricted yellow
spread yellow

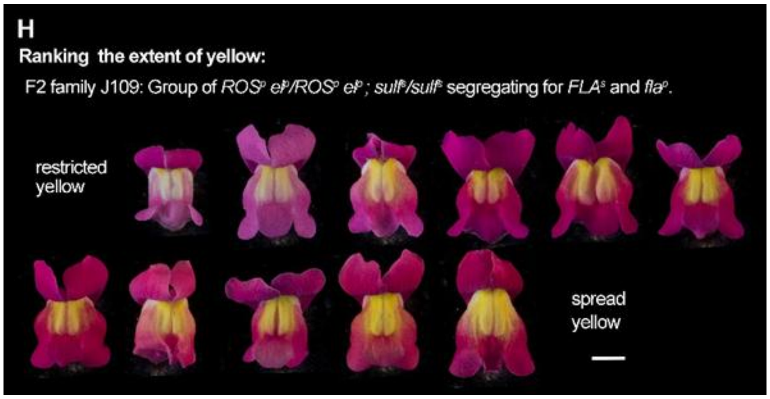
H
Ranking the extent of yellow:
F2 family J109: Group of *ROS*^{p} *el*^{p}/*ROS*^{p} *el*^{p} ; *sulf*^{s}/*sulf*^{s} segregating for *FLA*^{s} and *fla*^{p}.
restricted yellow
spread yellow

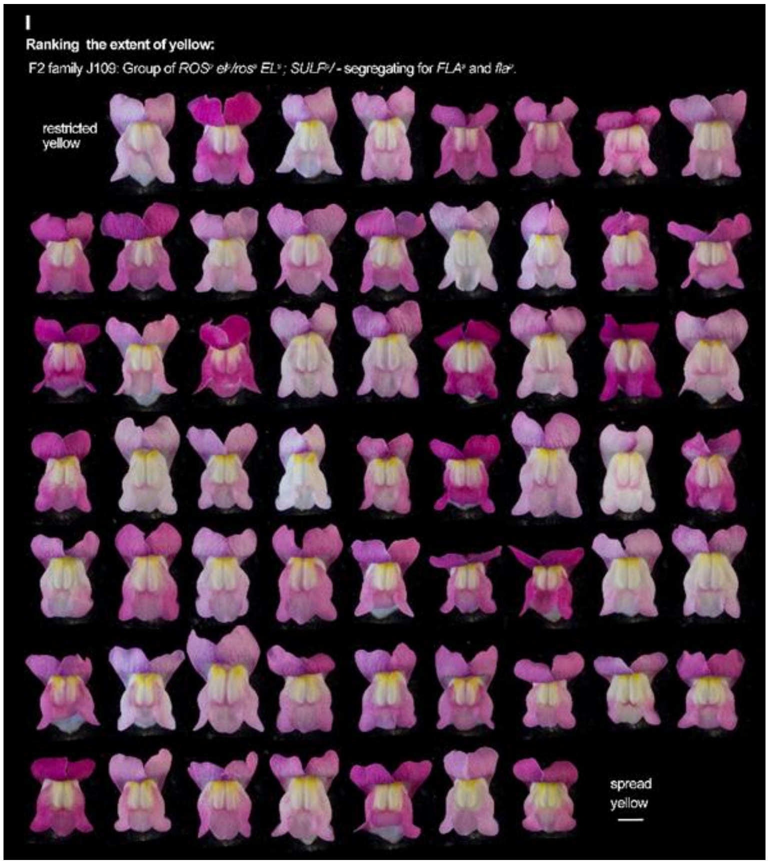
I
Ranking the extent of yellow:
F2 family J109: Group of ROS^p el^p/ros^s EL^s; SULF^p/- segregating for FLA^s and fla^p.
restricted yellow
spread yellow

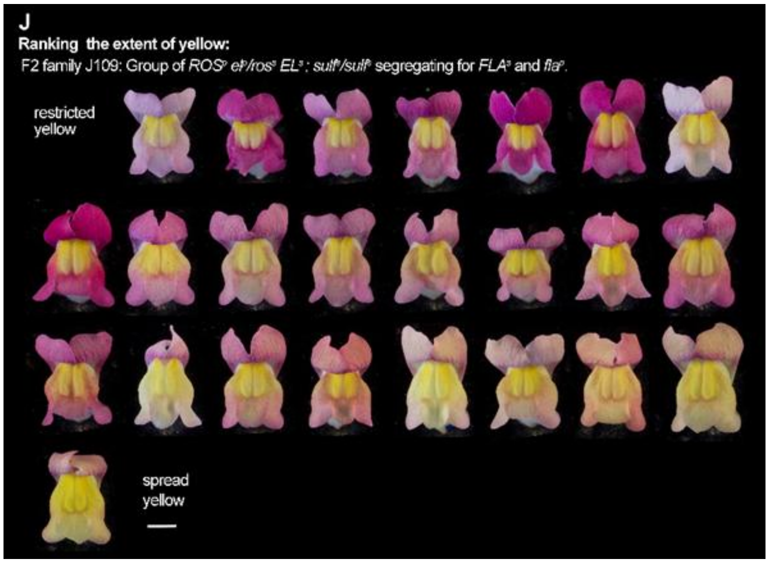
J
Ranking the extent of yellow:
F2 family J109: Group of ROS^p el^p/ros^s EL^s; sulf^s/sulf^s segregating for FLA^s and fla^p.
restricted yellow
spread yellow

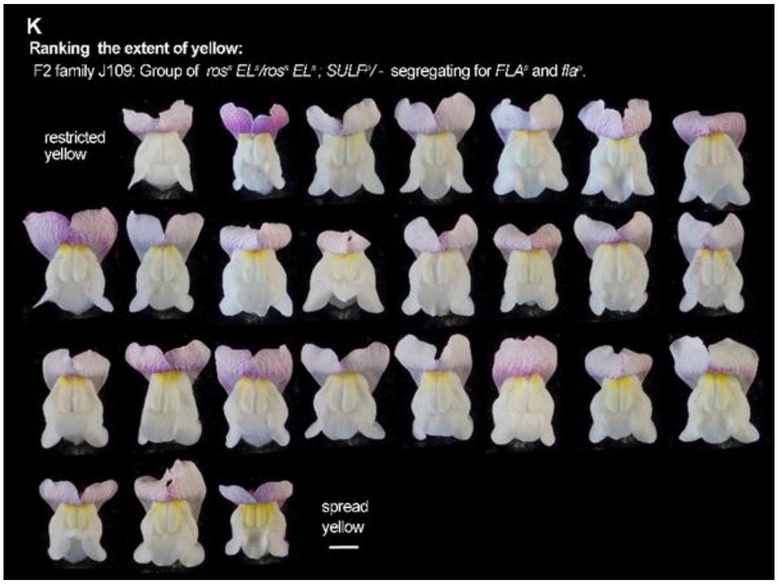
K
Ranking the extent of yellow:
F2 family J109: Group of ros^{s} EL^{s}/ros^{s} EL^{s}; $SULF^{p}$/- segregating for FLA^{s} and fla^{p}.
restricted yellow
spread yellow

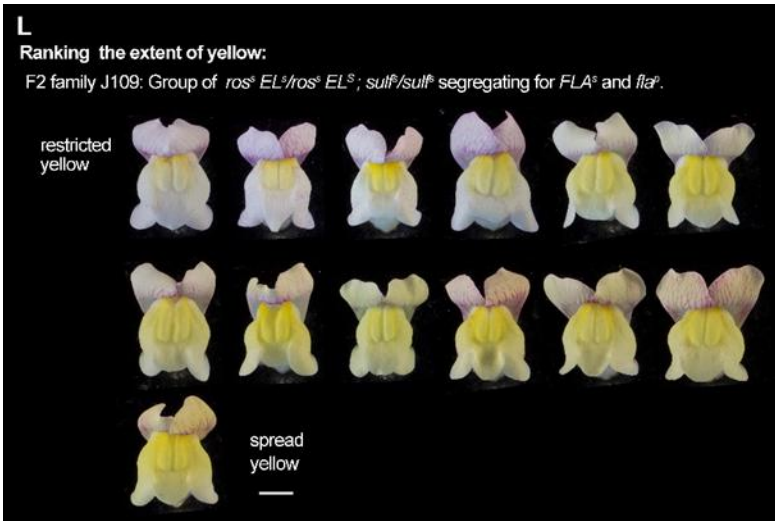
L
Ranking the extent of yellow:
F2 family J109: Group of ros^{s} EL^{s}/ros^{s} EL^{S}; $sulf^{s}$/$sulf^{s}$ segregating for FLA^{s} and fla^{p}.
restricted yellow
spread yellow

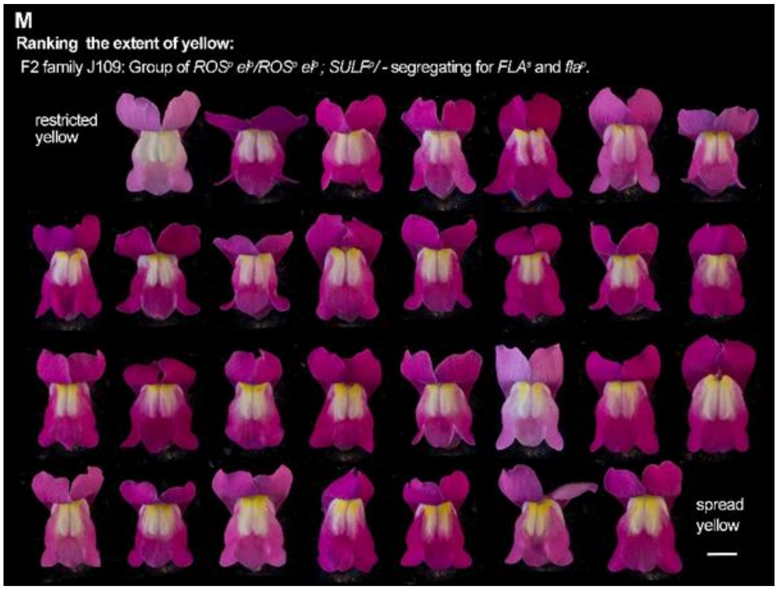
M
Ranking the extent of yellow:
F2 family J109: Group of ROS^{p} el^{p}/ROS^{p} el^{p} ; $SULF^{p}$/ - segregating for FLA^{s} and fla^{p}.
restricted yellow
spread yellow

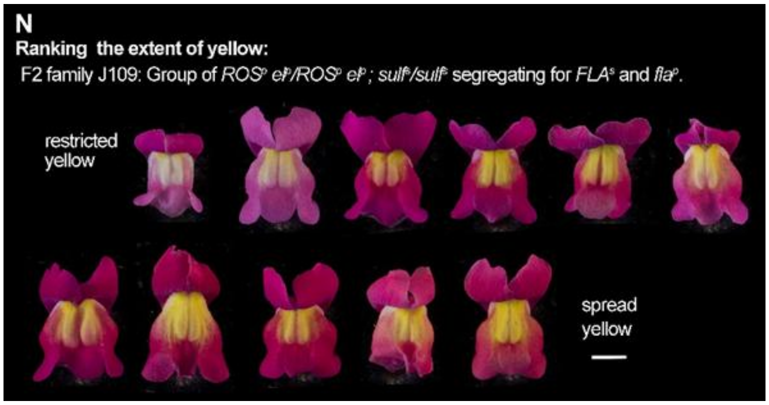
N
Ranking the extent of yellow:
F2 family J109: Group of ROS^{p} el^{p}/ROS^{p} el^{p} ; $sulf^{s}$/$sulf^{s}$ segregating for FLA^{s} and fla^{p}.
restricted yellow
spread yellow

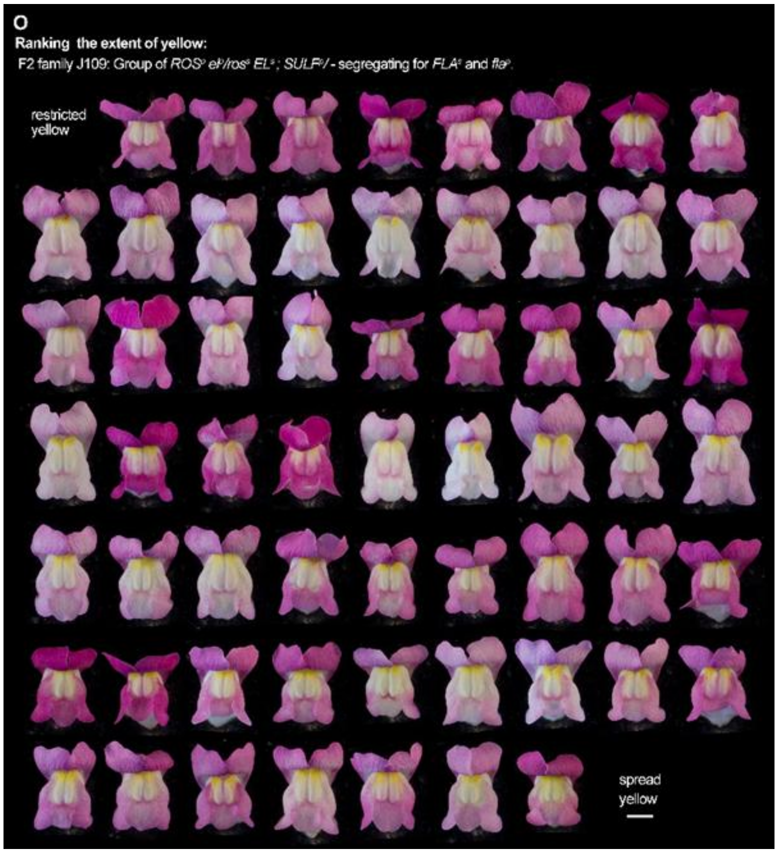
O
Ranking the extent of yellow:
F2 family J109: Group of ROS^{p} el^{p}/ros^{s} EL^{s} ; $SULF^{p}/-$ segregating for FLA^{s} and fla^{p}.
restricted yellow
spread yellow

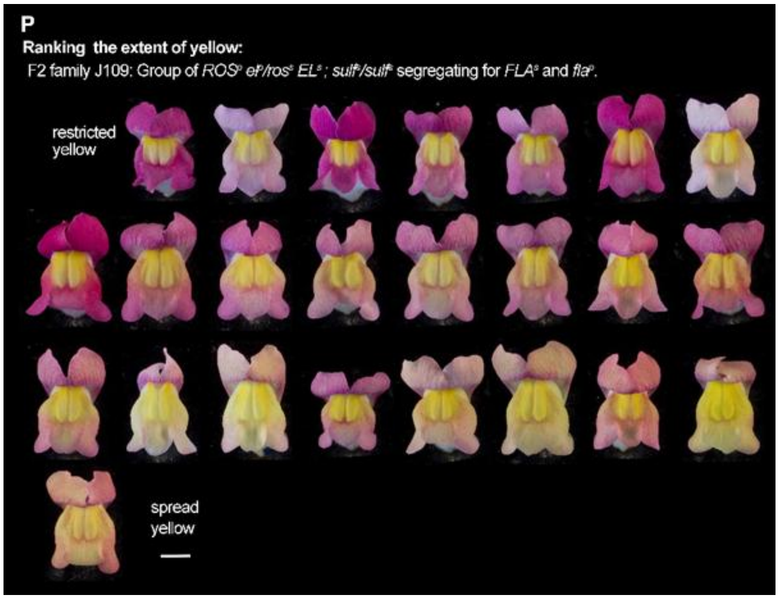
P
Ranking the extent of yellow:
F2 family J109: Group of ROS^{p} el^{p}/ros^{s} EL^{s} ; $sulf^{s}/sulf^{s}$ segregating for FLA^{s} and fla^{p}.
restricted yellow
spread yellow

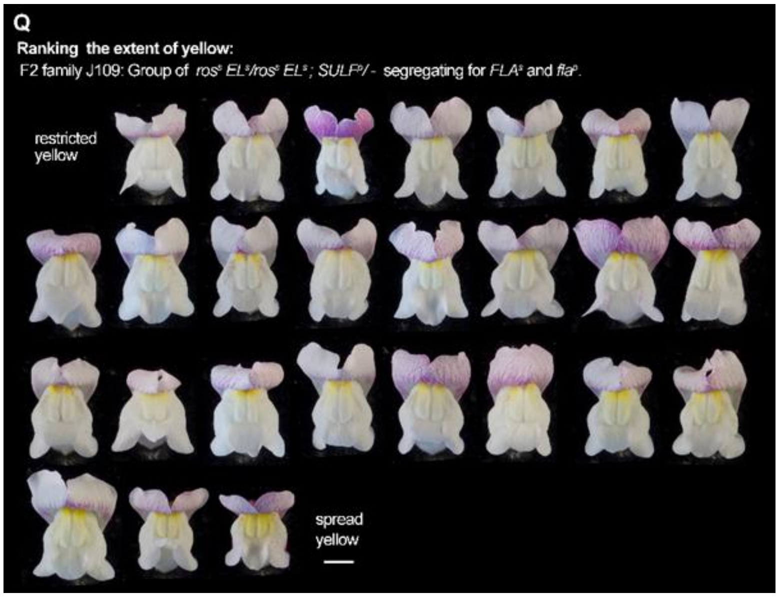
Q
Ranking the extent of yellow:
F2 family J109: Group of ros^{s} EL^{s}/ros^{s} EL^{s}; $SULF^{p}$/- segregating for FLA^{s} and fla^{p}.
restricted yellow
spread yellow

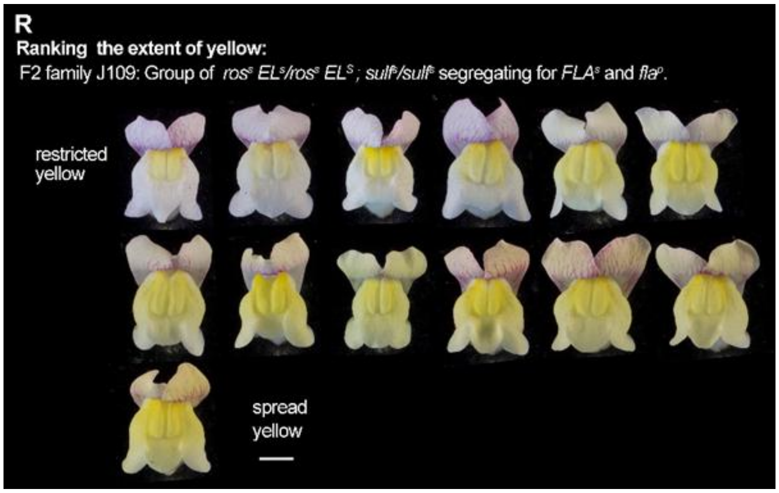
R
Ranking the extent of yellow:
F2 family J109: Group of ros^{s} EL^{s}/ros^{s} EL^{S}; $sulf^{s}$/$sulf^{s}$ segregating for FLA^{s} and fla^{p}.
restricted yellow
spread yellow

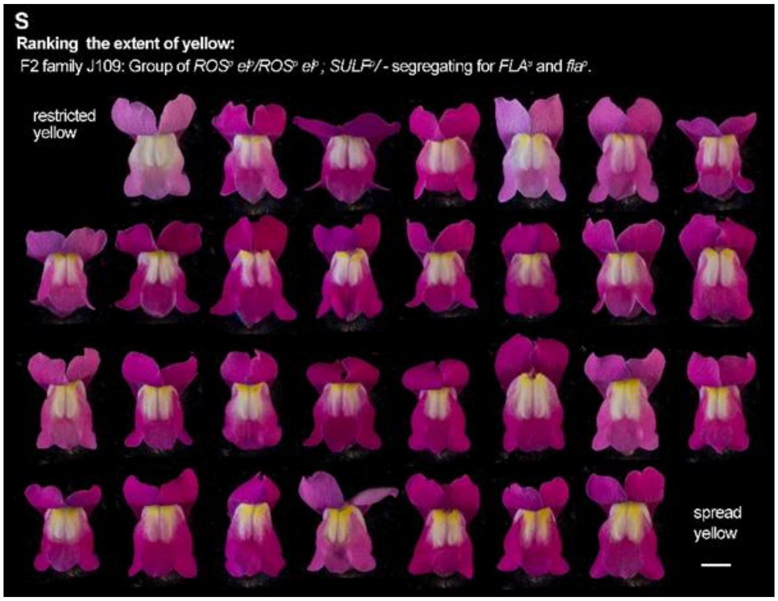
S
Ranking the extent of yellow:
F2 family J109: Group of ROS^p el^p/ROS^p el^p ; SULF^p/ - segregating for FLA^s and fla^p.
restricted yellow
spread yellow

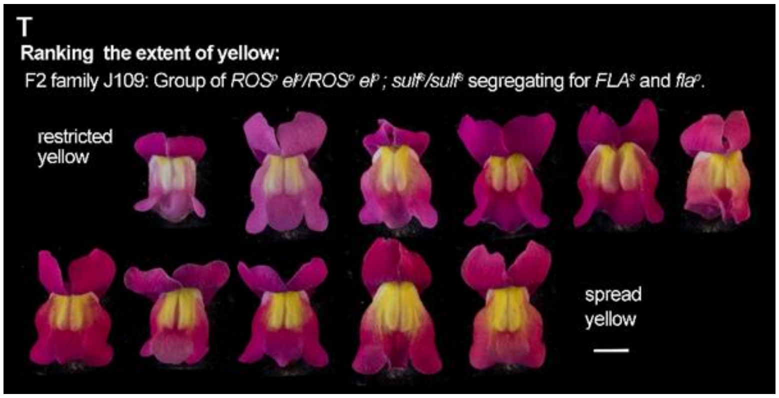
T
Ranking the extent of yellow:
F2 family J109: Group of ROS^p el^p/ROS^p el^p ; sulf^s/sulf^s segregating for FLA^s and fla^p.
restricted yellow
spread yellow

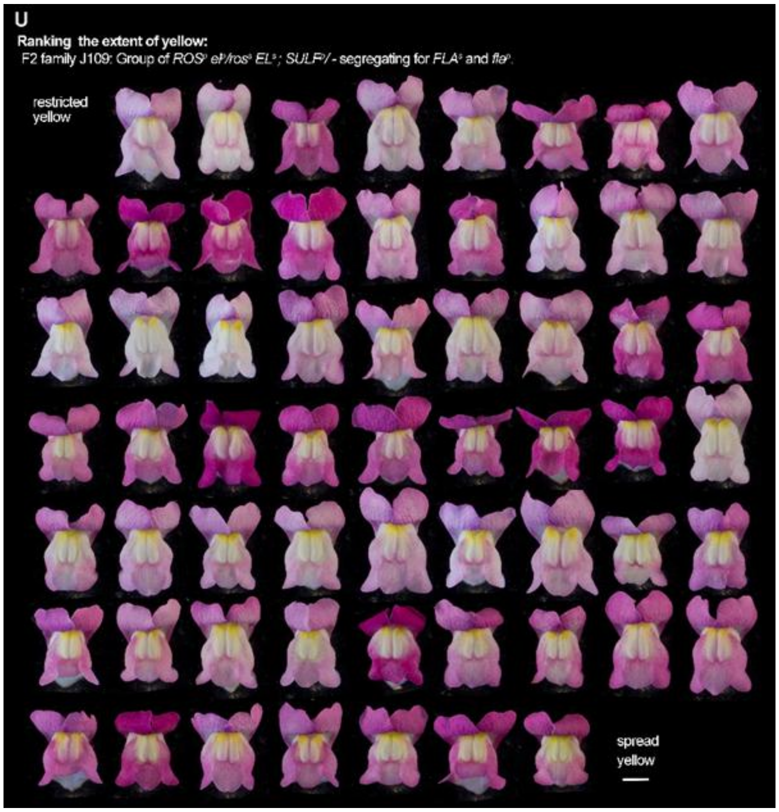
U
Ranking the extent of yellow:
F2 family J109: Group of ROS^{P} el^{P}/ros^{S} EL^{S}; $SULF^{P}$/- segregating for FLA^{S} and fla^{P}.
restricted yellow
spread yellow

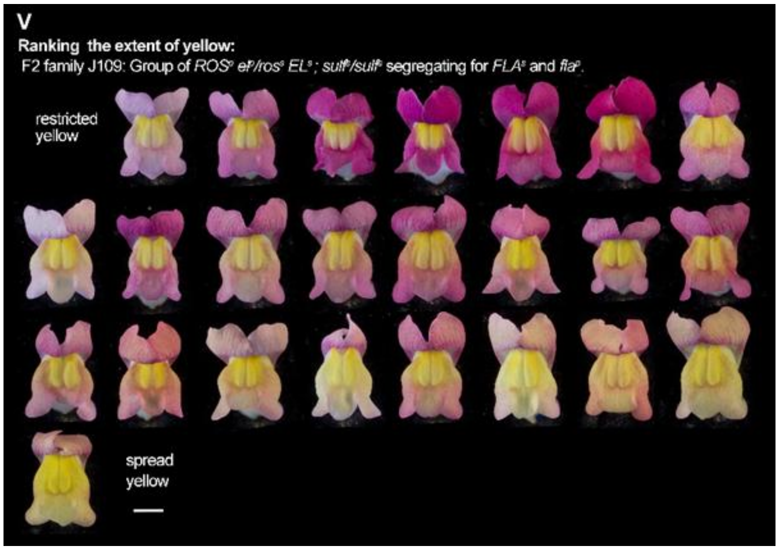
V
Ranking the extent of yellow:
F2 family J109: Group of ROS^{P} el^{P}/ros^{S} EL^{S}; $sulf^{S}$/$sulf^{S}$ segregating for FLA^{S} and fla^{P}.
restricted yellow
spread yellow

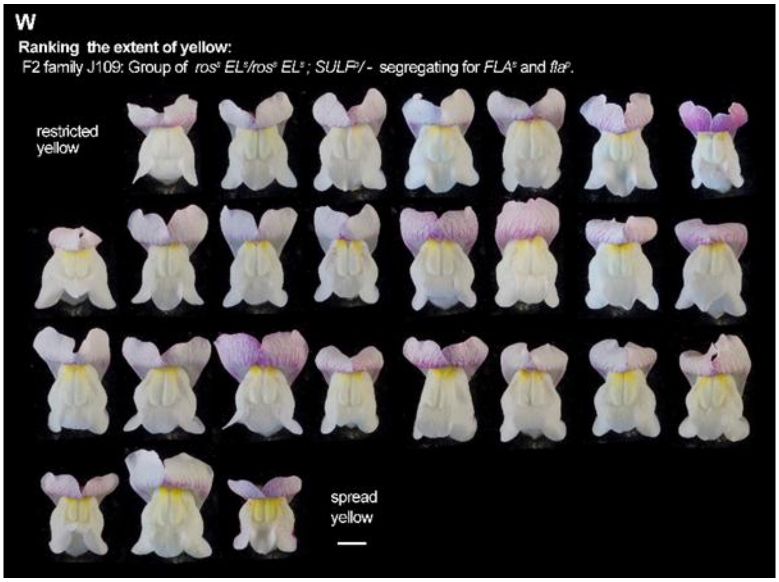
W
Ranking the extent of yellow:
F2 family J109: Group of ros^s EL^s/ros^s EL^s; $SULF^p/-$ segregating for FLA^s and fla^p.
restricted yellow
spread yellow

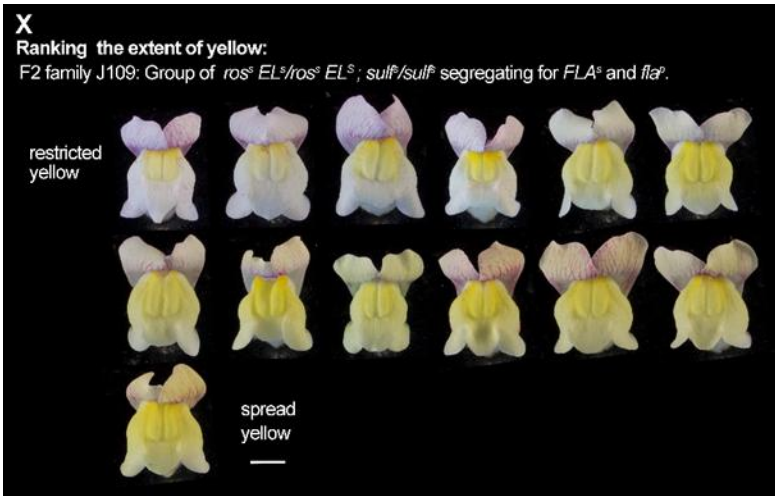
X
Ranking the extent of yellow:
F2 family J109: Group of ros^s EL^s/ros^s EL^S; $sulf^s/sulf^s$ segregating for FLA^s and fla^p.
restricted yellow
spread yellow

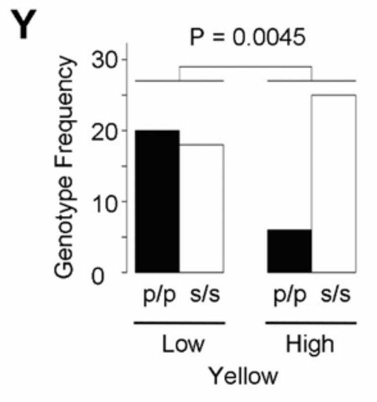
Y
P = 0.0045
Genotype Frequency
30
20
10
0
p/p s/s
p/p s/s
Low
High
Yellow

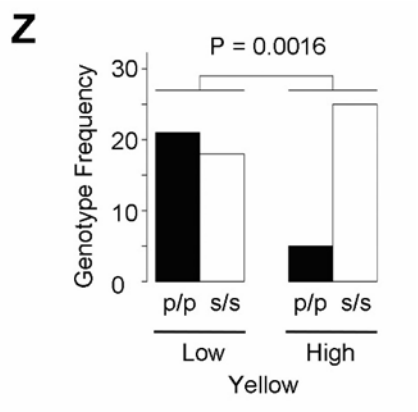
Z
P = 0.0016
Genotype Frequency
30
20
10
0
p/p s/s
p/p s/s
Low
High
Yellow

Fig. S3. Flowers ranked by extent of colour: family J109 (F2 of *A.m.m.* var. *pseudomajus* x *A.m.m.* var. *striatum*).

(**A-F**) Six different genotyped groups, where FLA^s and fla^p were segregating, were separately ranked for the extent of their magenta pigmentation. (**G-L**). The same groups were also ranked for their extent of yellow pigmentation and results analysed as for magenta. In each case, rankings were made without knowing the *FLA* genotypes. Three researchers, Daniel, Desmond and Tingting, made independent rankings for yellow: Daniel (G-L), Desmond (**M-R**) and Tingting (**S-X**). Similar results were found for all 3 yellow rankings: Daniel data (see Fig.2), Desmond (**Y**) and Tingting (**Z**). Each genotype group was ranked separately and the results then aggregated for all classes. The high yellow phenotypes were significantly enriched for s/s homozygotes with chi-squared P values shown.

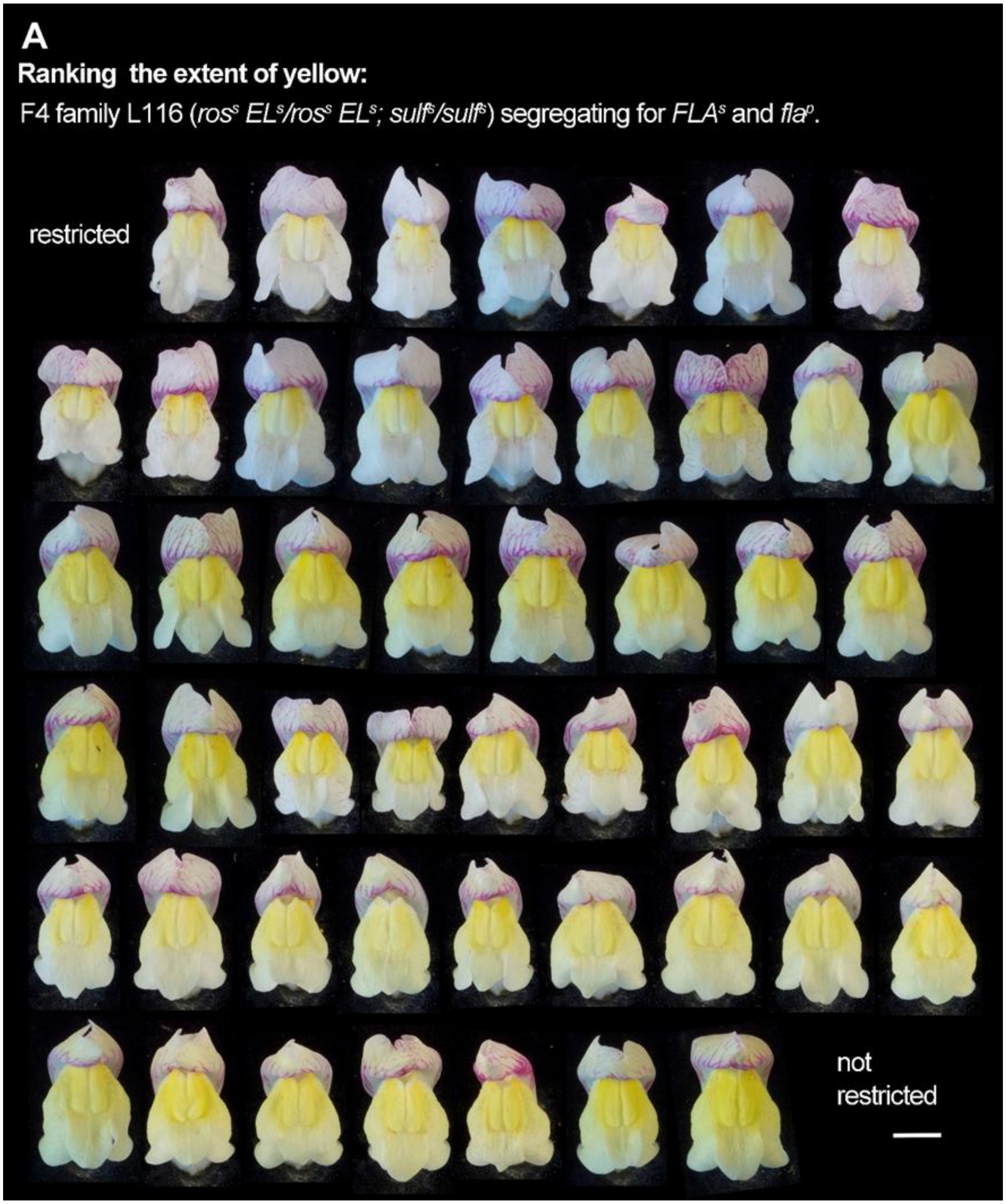
A
Ranking the extent of yellow:
F4 family L116 (ros^s EL^s/ros^s EL^s; $sulf^s$/$sulf^s$) segregating for FLA^s and fla^p.
restricted
not restricted

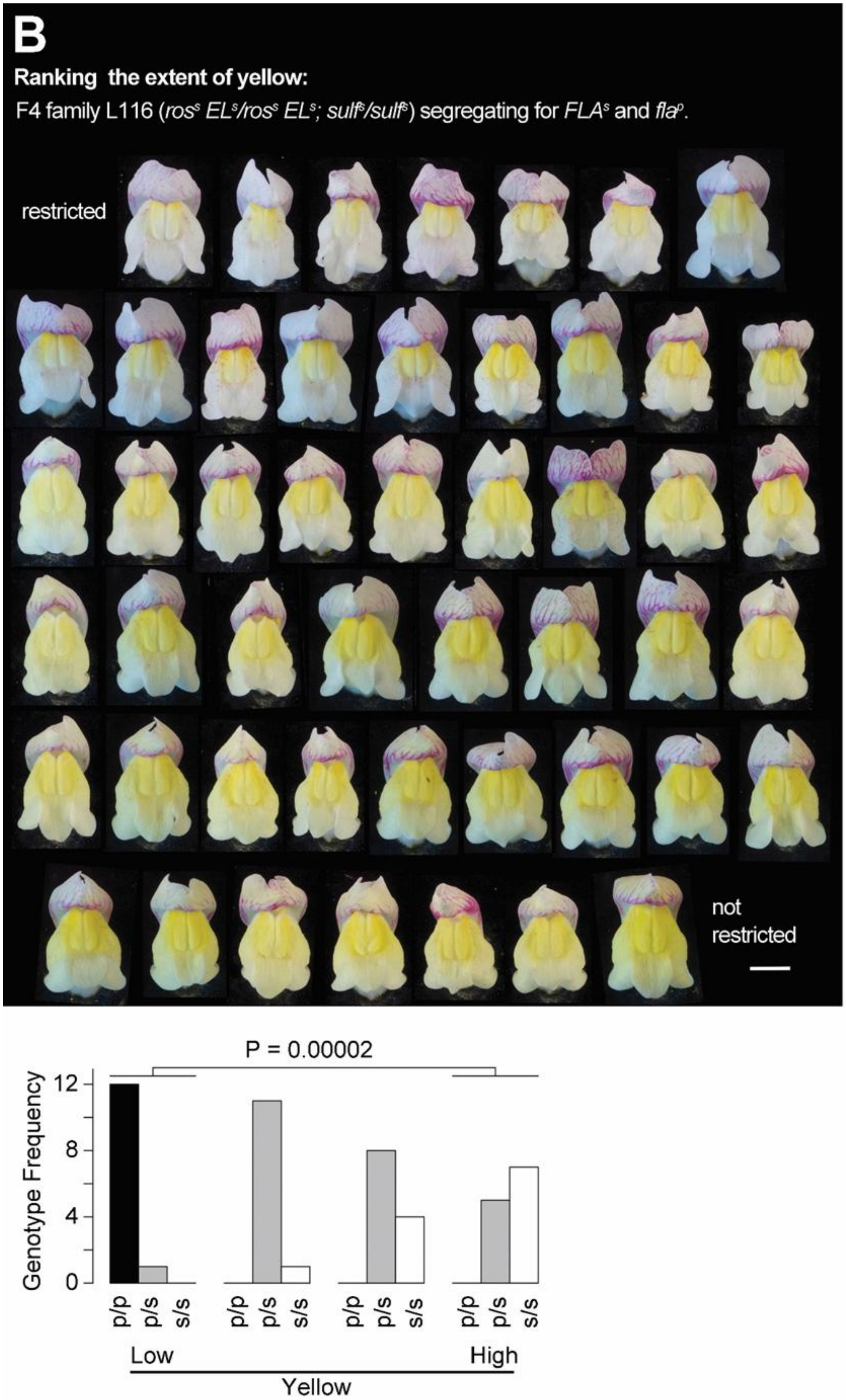
B
Ranking the extent of yellow:
F4 family L116 (ros^s EL^s/ros^s EL^s; $sulf^s$/$sulf^s$) segregating for FLA^s and fla^p.
restricted
not restricted
P = 0.00002
Genotype Frequency
0
4
8
12
p/p
p/s
s/s
Low
High
Yellow

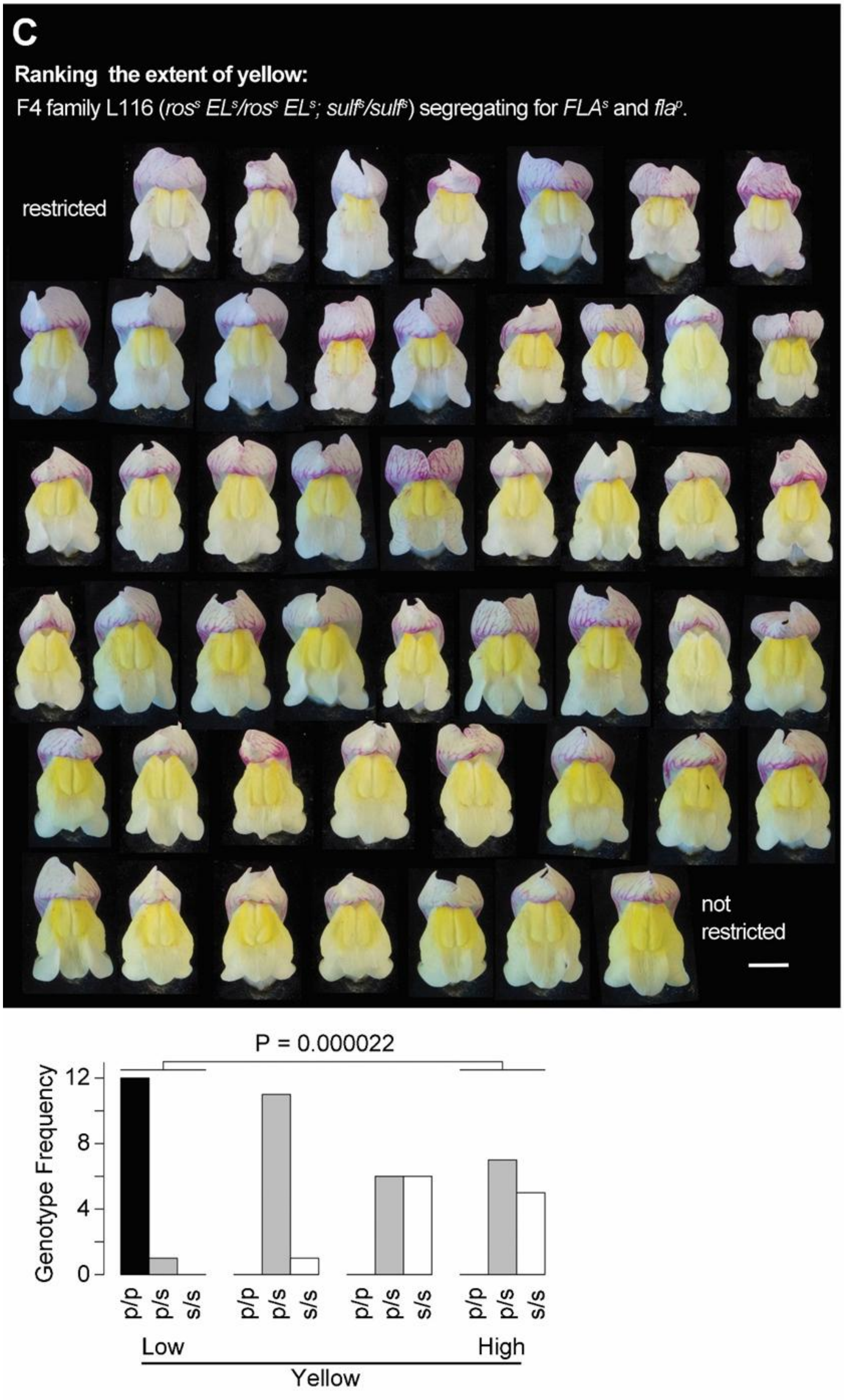
C
Ranking the extent of yellow:
F4 family L116 (ros^s EL^s/ros^s EL^s; sulf^s/sulf^s) segregating for FLA^s and fla^p.
restricted
not restricted
P = 0.000022
Genotype Frequency
12
8
4
0
p/p p/s s/s p/p p/s s/s p/p p/s s/s p/p p/s s/s
Low
High
Yellow

Fig. S4. Flowers ranked for yellow colour: F4 family L116 (*ros^s EL^s/ ros^s EL^s sulf^s/sulf^s*). Family L116 was segregating for *FLA^s* and *fla^p*. (**A**) A flower from each individual plant was ranked for its extent of yellow throughout its petal lobes. The ranked plants were grouped into 4 quartiles, their genotypes determined and plotted in Fig.2C. (**B**) Ranked flowers and graphed data from a second person. (**C**) Ranked flowers and graphed data from a third person.

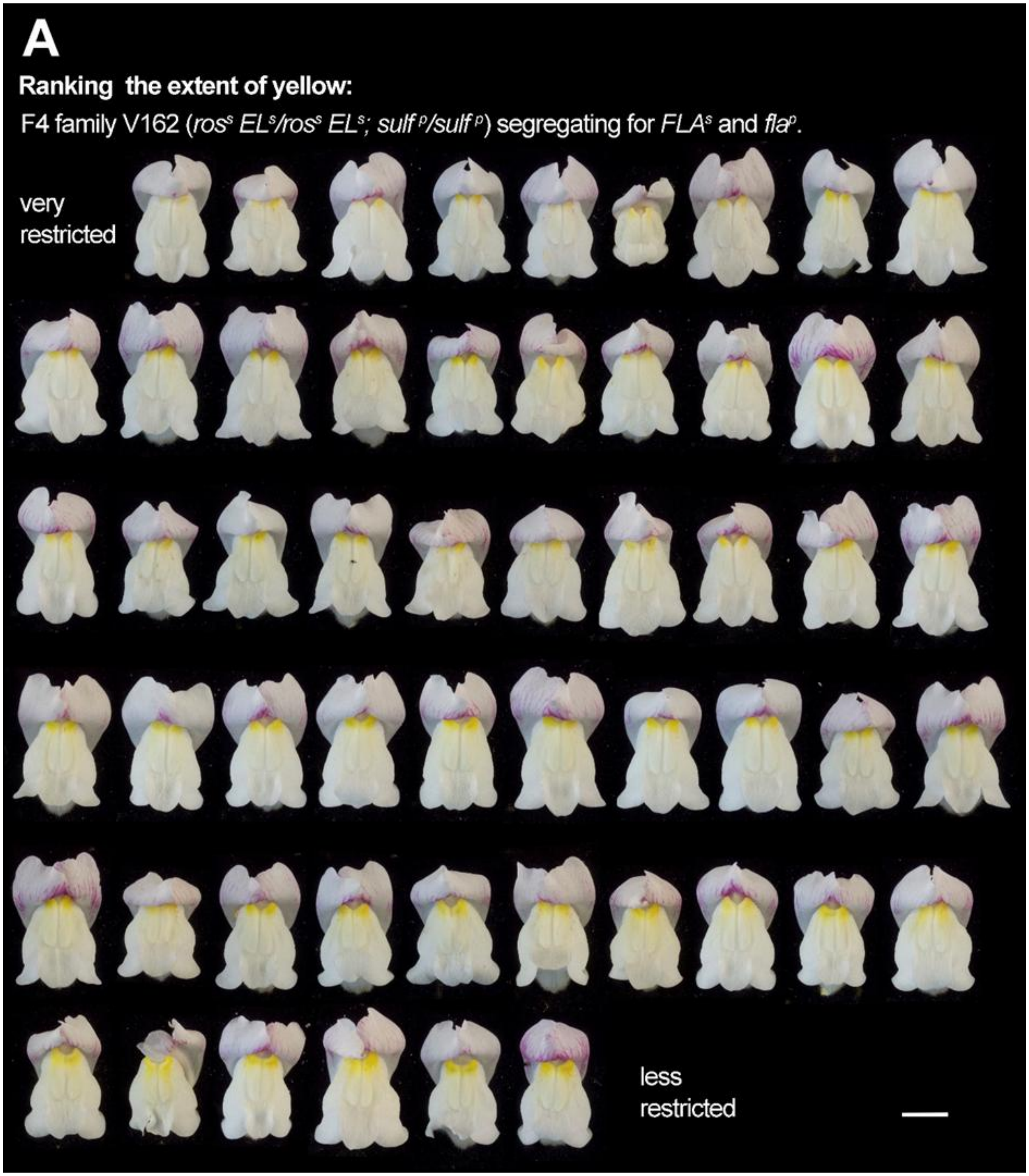
A
Ranking the extent of yellow:
F4 family V162 (ros^s EL^s/ros^s EL^s; sulf^p/sulf^p) segregating for FLA^s and fla^p.
very
restricted
less
restricted

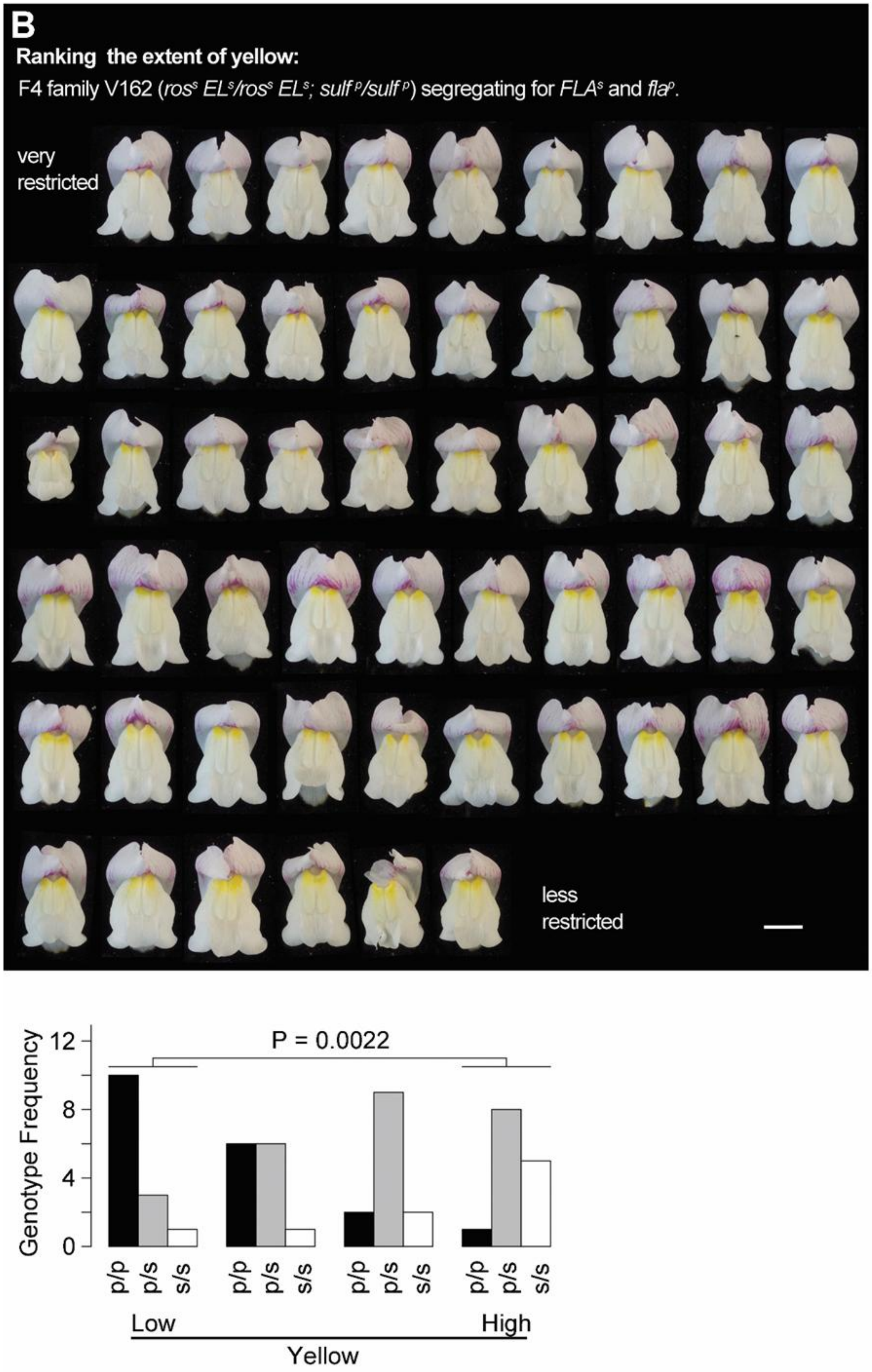
B
Ranking the extent of yellow:
F4 family V162 (*ros*^{s} *EL*^{s}/*ros*^{s} *EL*^{s}; *sulf*^{p}/*sulf*^{p}) segregating for *FLA*^{s} and *fla*^{p}.
very restricted
less restricted
P = 0.0022
Genotype Frequency
12
8
4
0
p/p
p/s
s/s
Low
High
Yellow

Fig. S5. Flowers ranked for yellow colour: F4 family V162 (*ros^s^ EL^s^/ ros^s^ EL^s^ SULF^p^/SULF^p^*).

Family V162 was segregating for *FLA^s^* and *fla^p^*. (**A**) A flower from each individual plant was ranked for its extent of yellow throughout its petal lobes. The ranked plants were grouped into 4 quartiles, their genotypes determined and plotted in Fig.2D. (**B**) A second person's independent ranking of flowers and graphed data as described in Fig.2D with chi-squared P value determined for lowest versus highest quartiles.

Ranking results from an F2 population (n=69) of *A.m.m.* var. *pseudomajus* (p) x *A.m.m.* var. *striatum* (s) ranked for extent of petal lobe magenta and genotyped for SNP2A. Individuals were grouped and ranked separately according to *ros*, *el*, and *sulf* genetic backgrounds, and results were aggregated across all genotypic classes. The low and high magenta categories have no significant difference in frequencies of p/p and s/s homozygotes (P values calculated using a contingency chi-squared test between the low and high quartiles). (B) Ranking the same population for extent of yellow shows s/s homozygotes significantly enriched in the high yellow category. (C) A *ros^s^ EL^s^/ros^s^ EL^s^ sulf^s^/sulf^s^* population genotyped for SNP2A and ranked for the extent of yellow. The lowest yellow quartile is significantly enriched for p/p homozygotes. Examples of flowers from the middle of each quartile are shown below. Bar is 1 cm. (D) A r*os^s^ EL^s^/ros^s^ EL^s^ SULF^p^/SULF^p^* population ranked for their extent of yellow. The lowest yellow quartile is significantly enriched for p/p homozygotes. Bar is 1 cm. Yellow rankings in B and C were done in triplicate by 3 independent observers and all gave similar significant P values (fig.S3).

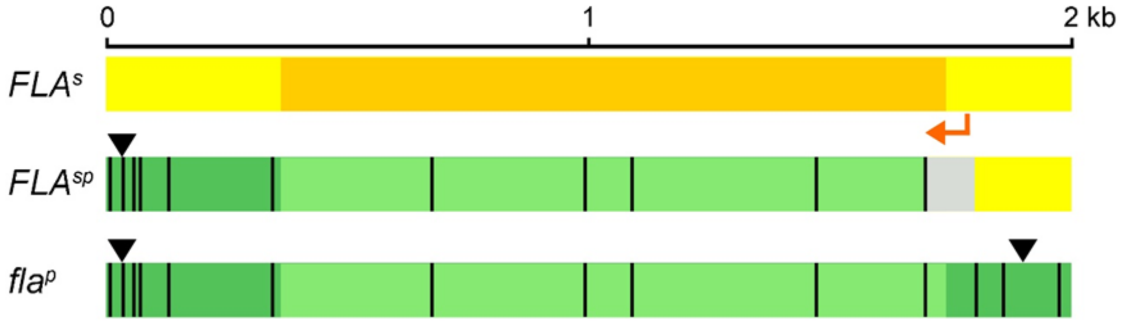

Fig. S6. Hybrid Zone Recombinant Structure.

The *FLA* region is shown for hybrid zone alleles of *A.m.m.* var. *striatum* (*FLA*^{s}), *A.m.m.* var. *pseudomajus* (*fla*^{p}) and a recombinant (*FLA*^{sp}) found at high frequency in the hybrid zone. The *FLA* coding region (different shade) is *fla*nked by ~300-400 bp of 5' sequence and 3' sequence, with SNPs (relative to *FLA*^{s}) marked as black lines, and indels as black triangles. The orange arrow indicates transcription direction. The hybrid zone recombination event has joined the 5' region of *FLA*^{s} to the coding and downstream region of *fla*^{p}. As the *FLA*^{s} and *fla*^{p} sequences are identical near the start region, the recombination point must lie between the two closest SNPs shown by the grey region.

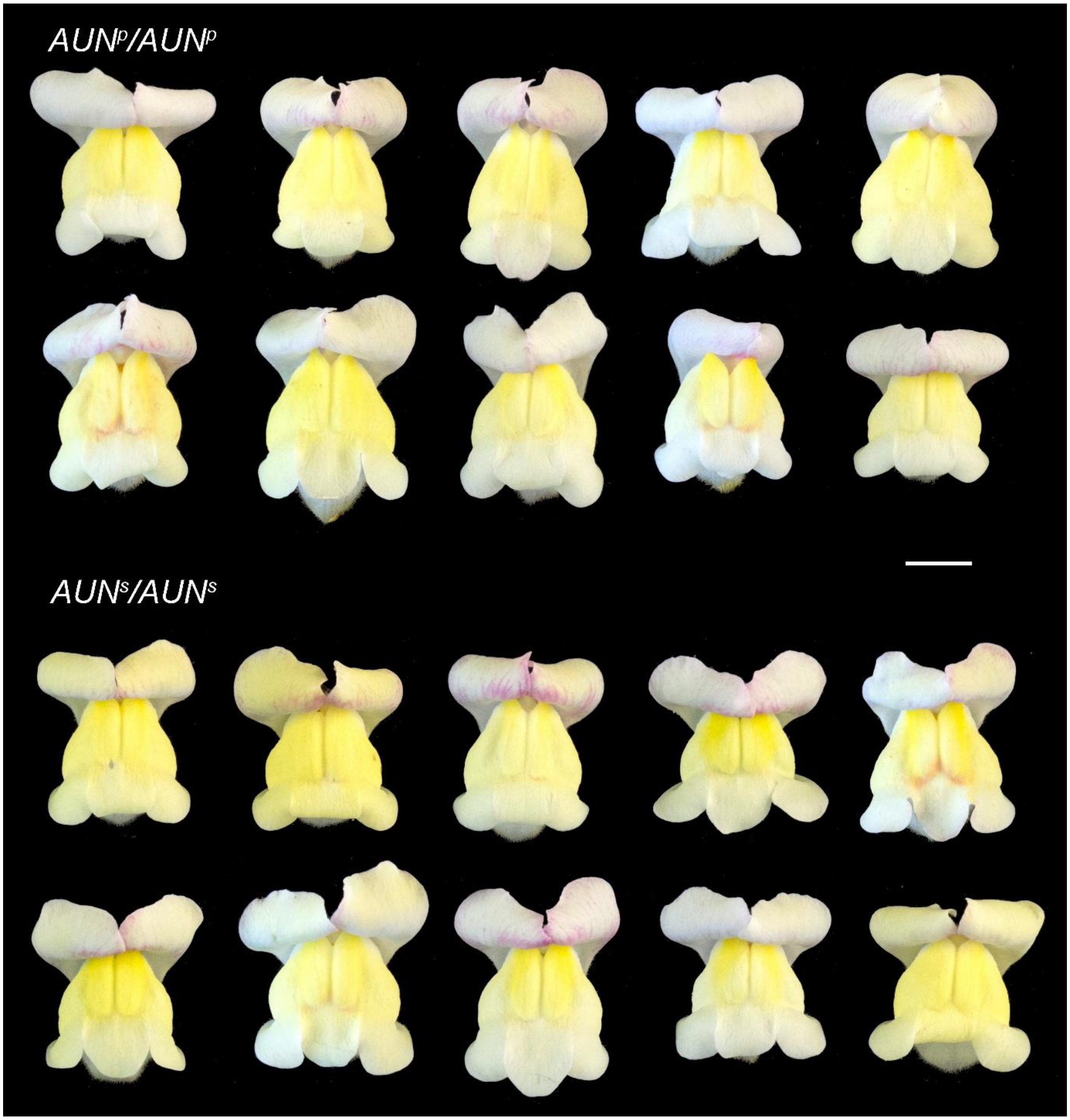

Fig. S7. Example flower photographs for AUN alleles.

Photographs of *AUN*^{s}*/AUN*^{s} and *AUN*^{p}*/AUN*^{p} from 10 different plants in a *sulf*^{s}*/sulf*^{s} *ros*^{s}*/ros*^{s} *FLA*^{s}*/FLA*^{s} *CRE*^{p}*/CRE*^{p} background. Scale bar (white line) = 1cm

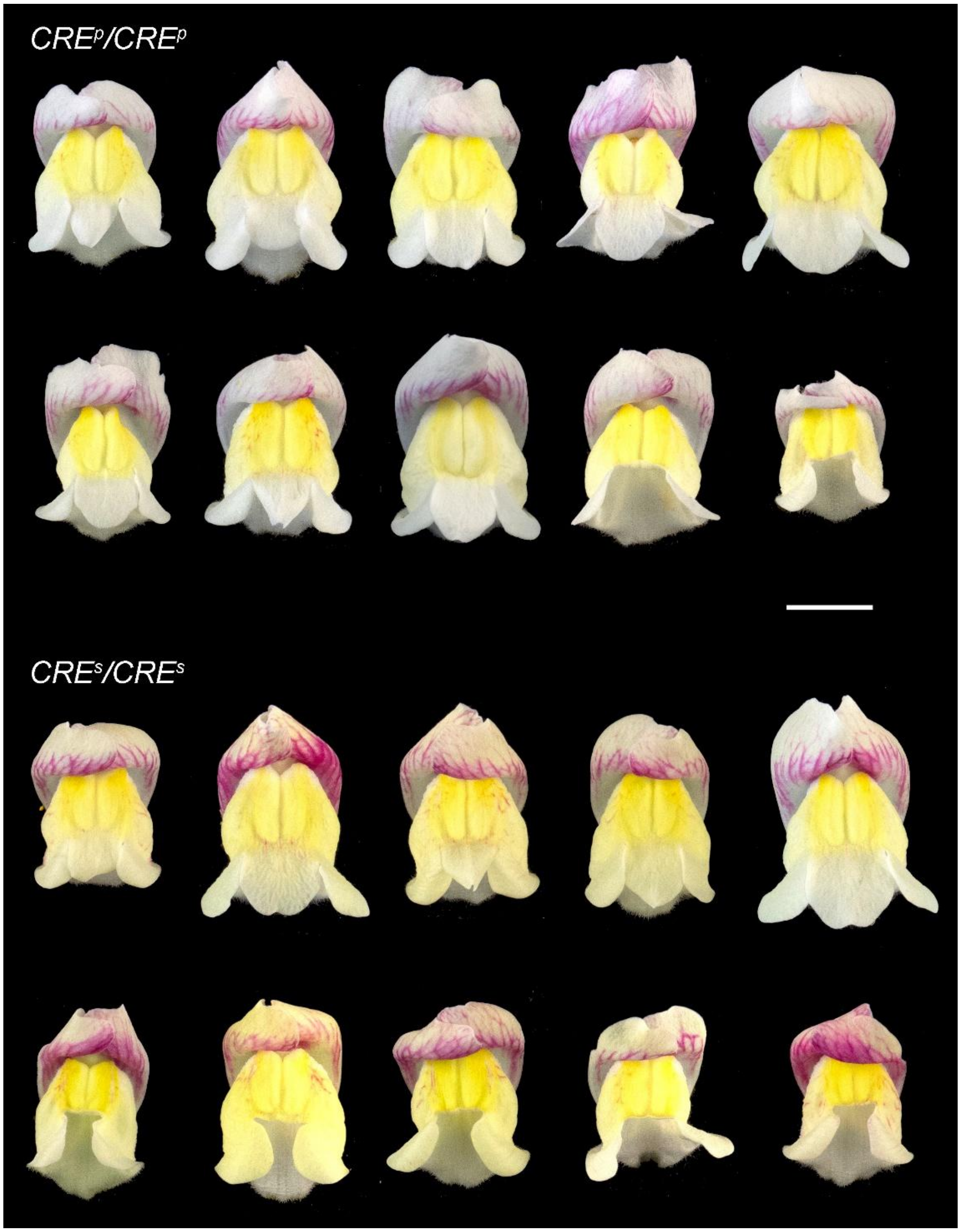

Fig. S8. Example flower photographs for *CRE* alleles.

Photographs of CRE^{p}/CRE^{p} and CRE^{s}/CRE^{s} from 10 different plants in a $sulf^{s}/sulf^{s}$ ros^{s}/ros^{s} FLA^{s}/fla^{p} AUN^{s}/AUN^{p} background. Scale bar (white line) = 1cm

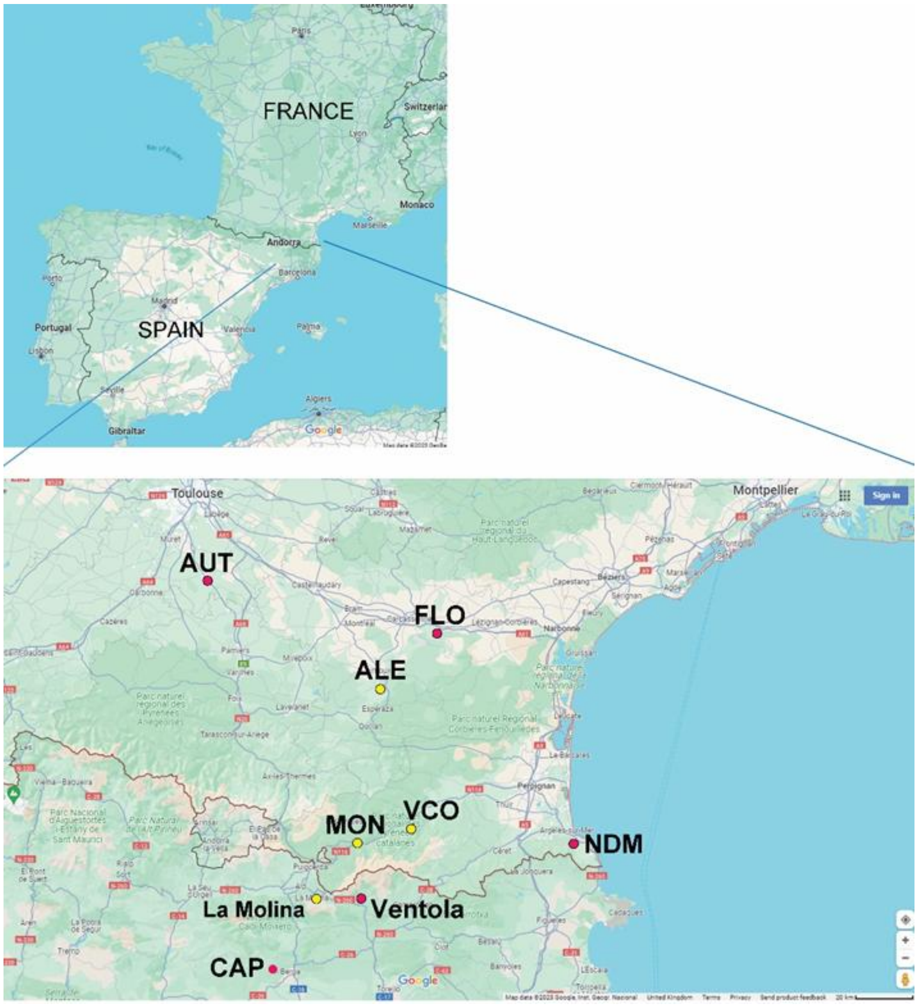

Fig. S9. Origin of *Antirrhinum* Species Populations Studied.

The wild *Antirrhinum* populations and Accessions used in this study were mapped to a region of south-west Europe. A close-up below, showed their distribution near the border between France and Spain. The Hybrid Zone details between La Molina and Ventola have been described by Tavares *et al*., 2018 (10).

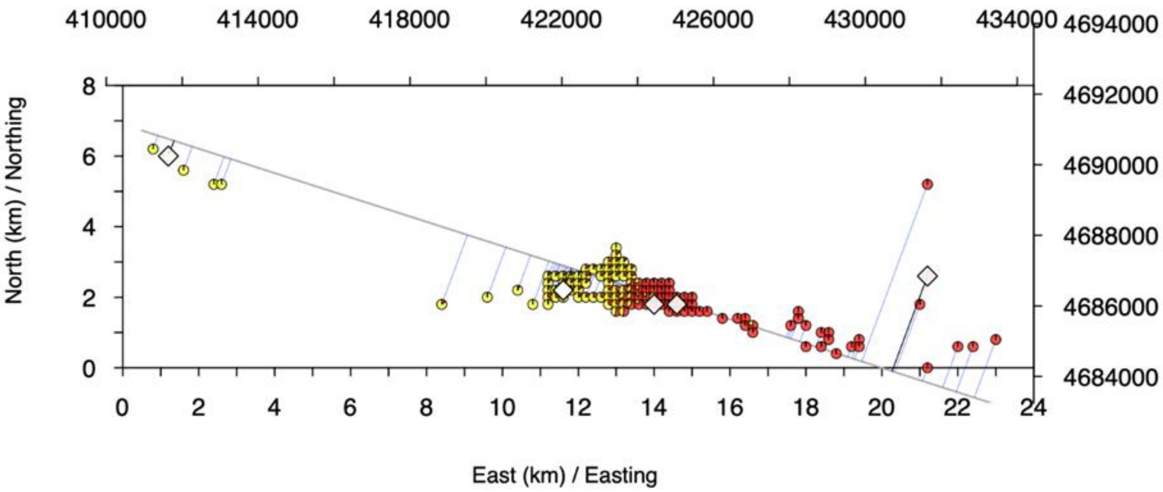

Fig. S10. Optimal Transect Fitting.

Allele frequencies (yellow = *A.m.m.* var. *striatum*, red = *A.m.m.* var. *pseudomajus*) indicated in pie charts at the *ROS1* locus in 200m demes. Each deme is collapsed to a geographic distance along the transect (black line) that is perpendicular (blue line) with the deme. Location of six Whole Genome PoolSeq demes shown as white diamonds. Distance along the hybrid zone show in kilometres and in Easting and Northings. Linear transect show is the optimal line chosen for cline fits with a gradient -0.345 and intercept at 6.9km.

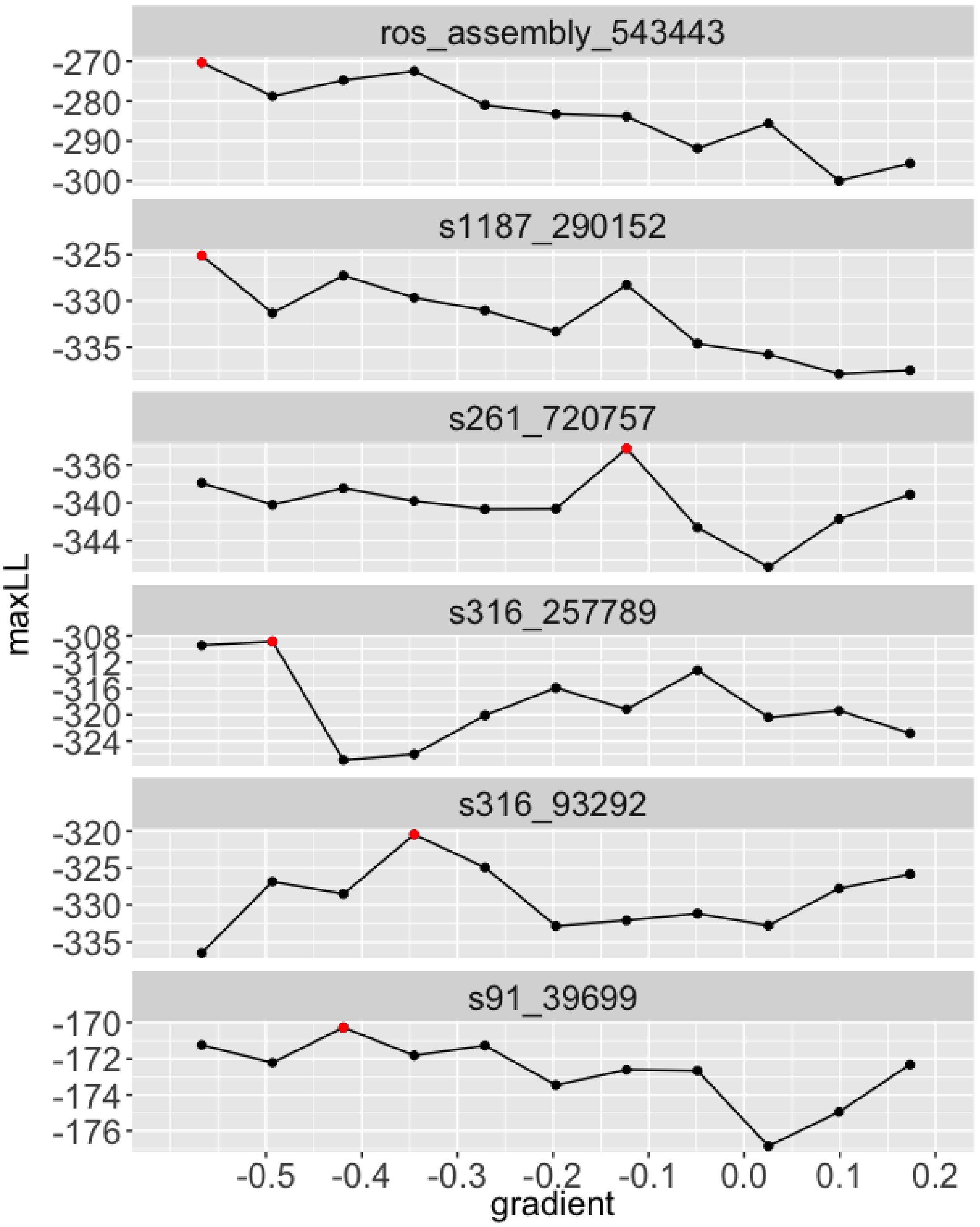

Fig. S11. Optimal Transects Across Loci.

Maximum log Liklihood (maxLL) for each parameter search at each transect directions (as gradients) through the hybrid zone at SNP markers at each of six key flower colour loci. The highest maxLL indicated with red filled circle.

A	B	C	D	E	F	G	H	I	J	K	L	M	N	O	P	Q	R	S	T	U	V	W	X	Y
hr2	POS	REF	ALT		D125	D186	D187	D151	D153	D155	D157	is Hor	is Hor	t	D169	D180	D181	D182	D183	D144	isHom	T		D292
hr2	53275016	C	A		HOM_REI	HOM_REI	HOM_REI	HOM_REI	HOM_REI	HOM_REI	HOM_REI	7	0		HOM_ALT	HOM_ALT	HOM_ALT	HOM_ALT	HOM_ALT	HOM_ALT	6			HOM_ALT
hr2	53275119	G	A		HOM_REI	HOM_REI	HOM_REI	HOM_REI	HOM_REI	HOM_REI	HOM_REI	7	0		HOM_ALT	HOM_ALT	HOM_ALT	HOM_ALT	HOM_ALT	HOM_ALT	6			HOM_ALT
hr2	53275152	C	T		HOM_REI	HOM_REI	HOM_REI	HOM_REI	HOM_REI	HOM_REI	HOM_REI	7	0		HOM_ALT	HOM_ALT	HOM_ALT	HOM_ALT	HOM_ALT	HOM_ALT	6			HOM_ALT
hr2	53275667	G	C		HOM_REI	HOM_REI	HOM_REI	HOM_REI	HOM_REI	HOM_REI	HOM_REI	7	0		HOM_ALT	HOM_ALT	HOM_ALT	HOM_ALT	HOM_ALT	HOM_ALT	6			HOM_ALT
hr2	53276008	G	C		HOM_REI	HOM_REI	HOM_REI	HOM_REI	HOM_REI	HOM_REI	HOM_REI	7	0		HOM_ALT	HOM_ALT	HOM_ALT	HOM_ALT	HOM_ALT	HOM_ALT	6			HOM_ALT
hr2	53276020	G	A		HOM_REI	HOM_REI	HOM_REI	HOM_REI	HOM_REI	HOM_REI	HOM_REI	7	0		HOM_ALT	HOM_ALT	HOM_ALT	HOM_ALT	HOM_ALT	HOM_ALT	6			HOM_ALT
hr2	53276027	C	A		HOM_REI	HOM_REI	HOM_REI	HOM_REI	HOM_REI	HOM_REI	HOM_REI	7	0		HOM_ALT	HOM_ALT	HOM_ALT	HOM_ALT	HOM_ALT	HOM_ALT	6			HOM_ALT
hr2	53276038	T	G		HOM_REI	HOM_REI	HOM_REI	HOM_REI	HOM_REI	HOM_REI	HOM_REI	7	0		HOM_ALT	HOM_ALT	HOM_ALT	HOM_ALT	HOM_ALT	HOM_ALT	6			HOM_ALT
hr2	53276052	T	G		HOM_REI	HOM_REI	HOM_REI	HOM_REI	HOM_REI	HOM_REI	HOM_REI	7	0		HOM_ALT	HOM_ALT	HOM_ALT	HOM_ALT	HOM_ALT	HOM_ALT	6			HOM_ALT
hr2	53276059	A	G		HOM_REI	HOM_REI	HOM_REI	HOM_REI	HOM_REI	HOM_REI	HOM_REI	7	0		HOM_ALT	HOM_ALT	HOM_ALT	HOM_ALT	HOM_ALT	HOM_ALT	6			HOM_ALT
hr2	53276061	G	A		HOM_REI	HOM_REI	HOM_REI	HOM_REI	HOM_REI	HOM_REI	HOM_REI	7	0		HOM_ALT	HOM_ALT	HOM_ALT	HOM_ALT	HOM_ALT	HOM_ALT	6			HOM_ALT
hr2	53276066	A	C		HOM_REI	HOM_REI	HOM_REI	HOM_REI	HOM_REI	HOM_REI	HOM_REI	7	0		HOM_ALT	HOM_ALT	HOM_ALT	HOM_ALT	HOM_ALT	HOM_ALT	6			HOM_ALT
hr2	53276128	A	T		HOM_REI	HOM_REI	HOM_REI	HOM_REI	HOM_REI	HOM_REI	HOM_REI	7	0		HOM_ALT	HOM_ALT	HOM_ALT	HOM_ALT	HOM_ALT	HOM_ALT	6			HOM_ALT
hr2	53276993	C	T		HOM_REI	HOM_REI	HOM_REI	HOM_REI	HOM_REI	HOM_REI	HOM_REI	7	0		HOM_ALT	HOM_ALT	HOM_ALT	HOM_ALT	HOM_ALT	HOM_ALT	6			HOM_ALT
hr2	53277087	C	T		HOM_REI	HOM_REI	HOM_REI	HOM_REI	HOM_REI	HOM_REI	HOM_REI	7	0		HOM_ALT	HOM_ALT	HOM_ALT	HOM_ALT	HOM_ALT	HOM_ALT	6			HOM_ALT
hr2	53277698	G	A		HOM_REI	HOM_REI	HOM_REI	HOM_REI	HOM_REI	HOM_REI	HOM_REI	7	0		HOM_ALT	HOM_ALT	HOM_ALT	HOM_ALT	HOM_ALT	HOM_ALT	6			HOM_ALT
hr2	53278320	T	C		HOM_REI	HOM_REI	HOM_REI	HOM_REI	HOM_REI	HOM_REI	HOM_REI	7	0		HOM_ALT	HOM_ALT	HOM_ALT	HOM_ALT	HOM_ALT	HOM_ALT	6			HOM_REF
hr2	53278347	G	A		HOM_REI	HOM_REI	HOM_REI	HOM_REI	HOM_REI	HOM_REI	HOM_REI	7	0		HOM_ALT	HOM_ALT	HOM_ALT	HOM_ALT	HOM_ALT	HOM_ALT	6			HOM_REF
hr2	53278482	C	A		HOM_REI	HOM_REI	HOM_REI	HOM_REI	HOM_REI	HOM_REI	HOM_REI	7	0		HOM_ALT	HOM_ALT	HOM_ALT	HOM_ALT	HOM_ALT	HOM_ALT	6			HOM_REF
hr2	53278560	G	T		HOM_REI	HOM_REI	HOM_REI	HOM_REI	HOM_REI	HOM_REI	HOM_REI	7	0		HOM_ALT	HOM_ALT	HOM_ALT	HOM_ALT	HOM_ALT	HOM_ALT	6			HOM_REF
hr2	53278573	G	A		HOM_REI	HOM_REI	HOM_REI	HOM_REI	HOM_REI	HOM_REI	HOM_REI	7	0		HOM_ALT	HOM_ALT	HOM_ALT	HOM_ALT	HOM_ALT	HOM_ALT	6			HOM_REF
hr2	53278574	T	A		HOM_REI	HOM_REI	HOM_REI	HOM_REI	HOM_REI	HOM_REI	HOM_REI	7	0		HOM_ALT	HOM_ALT	HOM_ALT	HOM_ALT	HOM_ALT	HOM_ALT	6			HOM_REF
hr2	53278957	A	T		HOM_REI	HOM_REI	HOM_REI	HOM_REI	HOM_REI	HOM_REI	HOM_REI	7	0		HOM_ALT	HOM_ALT	HOM_ALT	HOM_ALT	HOM_ALT	HOM_ALT	6			HOM_REF
hr2	53279039	T	A		HOM_REI	HOM_REI	HOM_REI	HOM_REI	HOM_REI	HOM_REI	HOM_REI	7	0		HOM_ALT	HOM_ALT	HOM_ALT	HOM_ALT	HOM_ALT	HOM_ALT	6			HOM_REF
hr2	53279055	G	T		HOM_REI	HOM_REI	HOM_REI	HOM_REI	HOM_REI	HOM_REI	HOM_REI	7	0		HOM_ALT	HOM_ALT	HOM_ALT	HOM_ALT	HOM_ALT	HOM_ALT	6			HOM_REF
hr2	53279075	G	C		HOM_REI	HOM_REI	HOM_REI	HOM_REI	HOM_REI	HOM_REI	HOM_REI	7	0		HOM_ALT	HOM_ALT	HOM_ALT	HOM_ALT	HOM_ALT	HOM_ALT	6			HOM_REF
hr2	53279076	T	A		HOM_REI	HOM_REI	HOM_REI	HOM_REI	HOM_REI	HOM_REI	HOM_REI	7	0		HOM_ALT	HOM_ALT	HOM_ALT	HOM_ALT	HOM_ALT	HOM_ALT	6			HOM_REF
hr2	53279085	A	T		HOM_REI	HOM_REI	HOM_REI	HOM_REI	HOM_REI	HOM_REI	HOM_REI	7	0		HOM_ALT	HOM_ALT	HOM_ALT	HOM_ALT	HOM_ALT	HOM_ALT	6			HOM_REF
hr2	53279199	A	C		HOM_REI	HOM_REI	HOM_REI	HOM_REI	HOM_REI	HOM_REI	HOM_REI	7	0		HOM_ALT	HOM_ALT	HOM_ALT	HOM_ALT	HOM_ALT	HOM_ALT	6			HOM_REF
hr2	53279259	A	T		HOM_REI	HOM_REI	HOM_REI	HOM_REI	HOM_REI	HOM_REI	HOM_REI	7	0		HOM_ALT	HOM_ALT	HOM_ALT	HOM_ALT	HOM_ALT	HOM_ALT	6			HOM_REF
hr2	53279260	T	C		HOM_REI	HOM_REI	HOM_REI	HOM_REI	HOM_REI	HOM_REI	HOM_REI	7	0		HOM_ALT	HOM_ALT	HOM_ALT	HOM_ALT	HOM_ALT	HOM_ALT	6			HOM_REF
hr2	53279279	T	G		HOM_REI	HOM_REI	HOM_REI	HOM_REI	HOM_REI	HOM_REI	HOM_REI	7	0		HOM_ALT	HOM_ALT	HOM_ALT	HOM_ALT	HOM_ALT	HOM_ALT	6			HOM_REF
hr2	53279321	G	A		HOM_REI	HOM_REI	HOM_REI	HOM_REI	HOM_REI	HOM_REI	HOM_REI	7	0		HOM_ALT	HOM_ALT	HOM_ALT	HOM_ALT	HOM_ALT	HOM_ALT	6			HOM_REF
hr2	53279349	A	G		HOM_REI	HOM_REI	HOM_REI	HOM_REI	HOM_REI	HOM_REI	HOM_REI	7	0		HOM_ALT	HOM_ALT	HOM_ALT	HOM_ALT	HOM_ALT	HOM_ALT	6			HOM_REF
hr2	53279389	C	A		HOM_REI	HOM_REI	HOM_REI	HOM_REI	HOM_REI	HOM_REI	HOM_REI	7	0		HOM_ALT	HOM_ALT	HOM_ALT	HOM_ALT	HOM_ALT	HOM_ALT	6			HOM_REF
hr2	53279459	C	G		HOM_REI	HOM_REI	HOM_REI	HOM_REI	HOM_REI	HOM_REI	HOM_REI	7	0		HOM_ALT	HOM_ALT	HOM_ALT	HOM_ALT	HOM_ALT	HOM_ALT	6			HOM_REF
hr2	53279481	A	T		HOM_REI	HOM_REI	HOM_REI	HOM_REI	HOM_REI	HOM_REI	HOM_REI	7	0		HOM_ALT	HOM_ALT	HOM_ALT	HOM_ALT	HOM_ALT	HOM_ALT	6			HOM_REF
hr2	53279926	T	A		HOM_REI	HOM_REI	HOM_REI	HOM_REI	HOM_REI	HOM_REI	HOM_REI	7	0		HOM_ALT	HOM_ALT	HOM_ALT	HOM_ALT	HOM_ALT	HOM_ALT	6			HOM_REF
hr2	53279936	C	T		HOM_REI	HOM_REI	HOM_REI	HOM_REI	HOM_REI	HOM_REI	HOM_REI	7	0		HOM_ALT	HOM_ALT	HOM_ALT	HOM_ALT	HOM_ALT	HOM_ALT	6			HOM_REF
hr2	53279953	C	G		HOM_REI	HOM_REI	HOM_REI	HOM_REI	HOM_REI	HOM_REI	HOM_REI	7	0		HOM_ALT	HOM_ALT	HOM_ALT	HOM_ALT	HOM_ALT	HOM_ALT	6			HOM_REF
hr2	53279971	G	A		HOM_REI	HOM_REI	HOM_REI	HOM_REI	HOM_REI	HOM_REI	HOM_REI	7	0		HOM_ALT	HOM_ALT	HOM_ALT	HOM_ALT	HOM_ALT	HOM_ALT	6			HOM_REF

Fig. S12. Diagnostic SNP across the *FLA* locus for Accessions of *striatum, pseudomajus* and a recombinant.

PCA was used to define a high confidence set of pure homozygotes (n=6 for *pseudomajus* [Accessions with Coen IDs D169, D180, D181, D182, D183, D144]) and n=7 for *striatum* [Accessions with Coen IDs D125, D186, D187, D151, D153, D155, D157]), with a recombinant Hybrid Zone line established with Coen ID D292. We called genotypes at biallelic SNVs across the interval 53.275-53.80Mb. Individual samples had a minimum depth of 5 and genotype quality of 15. The resulting 358 SNVs were filtered to show those that were diagnostically different between the two "pure" groups (n=41). Each row is a SNP, colour is according to genotype: yellow = HOM REF (*striatum*), magenta= HOM ALT (*pseudomajus*). The pure yellow and magenta columns are the two "diagnostic groupings", the next is the *FLA* homozygous recombinant. The two SNPs defining this breakpoint were mapped to *A.m. majus* genome v3 at Chr2: 53277698 and Chr2: 53278320, an interval of 622bp.

Table S1. Loci and Alleles used in this study.

Allele	Molecular Nature	Phenotype of alelle relative to that conferred by the ***straitum*** allele
AUN ^{P}	enzyme for yellow aurone synthesis (AS1)	yellow is reduced across petal
CRE ^{P}	unknown (candidates; O-methyltransferase or SCR-like TF)	yellow is reduced across petal
fla ^{P}	enzyme for yellow aurone synthesis (4'CGT)	yellow is restricted to proximal region of petal
FLA ^{SP}	recombinant allele of striatum promoter with pseudo coding	yellow is increased across petal
SULF ^{P}	long noncoding RNA generates small RNA against *FLA*	yellow is reduced in distal region of petal

Table S2. Oligos used in this study.

LOCUS	Marker	Marker	Other marker	Oligo	Oligo sequence	Oligo Position (A. majus v4)
	Type	Name	Name(s)	Name		
FLAVIA	KASP	s316-3	5'-FLA prom	#2205	GATTCCTCAAGCAGAAAACG	Chr2:53,661,809-53,661,828
(FLA)			s316_26294	#2206	GATTCCTCAAGCAGAAAACA	Chr2:53,661,809-53,661,828
			SNP2A	#2207	GGAGTGCATCCCTGCCGCG	Chr2:53,661,872-53,661,854
	KASP	s316-R2	3'-FLA down	do253	TTCACGTTCTACGAAGGGGTA	Chr2:53,437,564-53,437,544
			s316_250709	do254	TTCACGTTCTACGAAGGGGTT	Chr2:53,437,564-53,437,544
				do255	CTTTGCCCGTTGCTTGAC	Chr2:53,437,513-53,437,530
	SANGER			do259	TGTTATACGTTTGCGACTCACGAGC	Chr2:53,467,866-53,467,890
				do460	GCAGAAGAACAAATTCATCTCCG	Chr2:53,470,017-53,469,995
SULFUREA	KASP	set 65	s91_122,561	do514	GCAAAATCTGCCCTTTTCCAACTT	Chr4:38,397,156-38,397,179
(SULF)				do515	GCAAAATCTGCCCTTTTCCAACTA	Chr4:38,397,156-38,397,179
				do516	ACTGATGTGAGCGCCGACTGAGC	Chr4:38,397,207-38,397,185
	KASP	set 66	s91_181,717	do517	GAATACCACTAAACGAGTGAATGA	Chr4:38,456,336-38,456,359
				do518	GAATACCACTAAACGAGTGAATGG	Chr4:38,456,336-38,456,359
				do519	CTGAATGTCTTCGAAAGGACAGTG	Chr4:38,456,396-38,456,374
AURINA	KASP	set 61		do502	TGGAGTCTTAGCGCTCGACACC	CHr2:1,052,509-1,052,488
(AUR)				do503	TGGAGTCTTAGCGCTCGACACA	CHr2:1,052,509-1,052,488
				do504	CAATACCACTACTCCTGAAGAGC	Chr2:1,052,428-1,052,450
CREMOSA	KASP	set 54 B		do467	GTGACTTGGGAGGAAGAATAATC	Chr1:955,163-955,185
(CRE)				do468	GTGACTTGGGAGGAAGAATAATA	Chr1:955,163-955,185
				do469	TTGGTGATTAAGGGGAAAGTGAC	Chr1:955,225-955,203
	AFLP	475-6		do475	GAGGCTAGGAAGAAAGGTTTGTCG	Chr1:954,562-954,585
				do476	CTAACATTGAGCCAAATATTTGCC	Chr1:955,658-955,635
ROS1	KASP	ROS1 int		#1911	CAACATTGACGTACGGTATTC	Chr6:52,887,667-52,887,647
				#1912	CAACATTGACGTACGGTATTT	Chr6:52,887,667-52,887,647
				#1483	TGGCATCAAGTTCCACACAGAGCAG	Chr6:52,887,481-52,887,505
ELUTA	AFLP	1615-6		#1615	CATTGTCATGACTCGTTCAACA	Chr6:53,063,047-53,063,068
				#1616	TTAAACTGAAAGGCAGGCAATC	Chr6:53,063,517-53,063,496

recombination analysis						
ps x str	KASP	Fla_set 23		do275	ACTTGGTTGTCCGCGAGAAGCT	Chr2:12,126,989-12,126,968
				do276	ACTTGGTTGTCCGCGAGAAGCC	Chr2:12,126,989-12,126,968
				do277	CACGGATAAAAAGCAAATCTGGC	Chr2:12,126,933-12,126,955
	KASP	Fla_set 36		do314	AATCACCGCATTTCTGGAAGCC	Chr2:46,439,115-46,439,094
				do315	AATCACCGCATTTCTGGAAGCT	Chr2:46,439,115-46,439,094
				do316	TGATGTTTGGACCTTTCTGAGCC	Chr2:46,439,065-46,439,087
	KASP	Fla_set 33		do305	GAGAAACATAAGCCGAGTGGCC	Chr2:53,067,851-53,067,872
				do306	GAGAAACATAAGCCGAGTGGCT	Chr2:53,067,851-53,067,872
				do307	TCTCCATGCTGAAACACACGTCG	Chr2:53,067,909-53,067,887
	KASP	Fla_set 43		do364	GATTTGGTTGAAGAAAACAGTGC	Chr2:53,469,477-53,469,499
				do365	GATTTGGTTGAAGAAAACAGTGA	Chr2:53,469,477-53,469,499
				do366	AAGCTTCTATATAATTAAGACGG	Chr2:53,469,528-53,469,506
	KASP	Fla_set 31		do299	GAAGCTGGAGGTTTAGCTCGAT	Chr2:53,594,752-53,594,773
				do300	GAAGCTGGAGGTTTAGCTCGAC	Chr2:53,594,752-53,594,773
				do301	GAGATTTGCTATCTGGTATTGGAG	Chr2:53,594,824-53,594,801
	KASP	Fla_set 42		do361	TTACACAACGTTTGTTCATACA	Chr2:53,711,760-53,711,739
				do362	TTACACAACGTTTGTTCATACT	Chr2:53,711,760-53,711,739
				do363	GTCATGTGATACTTGTTGATCTGC	Chr2:53,711,709-53,711,732
	KASP	Fla_set 44		do367	ATAGAAGAGGCCCTTACCTAGG	Chr2:54,657,411-54,657,432
				do368	ATAGAAGAGGCCCTTACCTAGA	Chr2:54,657,411-54,657,432
				do369	AAAGTCCTAAGATACCTCAAAGC	Chr2:54,657,458-54,657,436
	KASP	Fla_set 30		do296	CACTTTGCCTTCTTTGAATATG	Chr2:59,512,733-59,512,712
				do297	CACTTTGCCTTCTTTGAATATA	Chr2:59,512,733-59,512,712
				do298	GCTCGAAAGGTTTAGTGATTGTC	Chr2:59,512,677-59,512,699
	KASP	Fla_set 29		do293	ATCCTTCATAGCTGATGAAATA	Chr2:63,046,469-63,046,448
				do294	ATCCTTCATAGCTGATGAAATG	Chr2:63,046,469-63,046,448
				do295	AGCTCCGAGCTGAATATGGAGCC	Chr2:63,046,414-63,046,436
	KASP	Fla_set 38		do320	ATCAAGTATATAACGAGATTGG	Chr2:72,179,984-72,179,963
				do321	ATCAAGTATATAACGAGATTGA	Chr2:72,179,984-72,179,963
				do322	TGGATCAGGAGAAGTATGTTC	Chr2:72,179,933-72,179,953

str x maj	KASP	Fla_set 22		do272	GGAAATGTTGTGCAACATTGGC	Chr2:5,356,724-5,356,745
				do273	GGAAATGTTGTGCAACATTGGG	Chr2:5,356,724-5,356,745
				do274	GGATCTTCATGGAAGGGCAACTC	Chr2:5,356,771-5,356,749
	KASP	Fla_set 26		do284	GCTCTCAAGTTTCAACCACACG	Chr2:41,257,084-41,257,063
				do285	GCTCTCAAGTTTCAACCACACA	Chr2:41,257,084-41,257,063
				do286	GTGTGAGGCAAGTTTCAAAAGAG	Chr2:41,257,026-41,257,048
	KASP	Fla_set 40		do326	AAAATACCGGGGAGAGAGAAGT	Chr2:57,901,885-57,901,906
				do327	AAAATACCGGGGAGAGAGAAGC	Chr2:57,901,885-57,901,906
				do328	CCTTCTCAATTGAAACCTTCACC	Chr2:57,901,930-57,901,908
	KASP	Fla_set 28		do290	GCAAATCTTGTTGTACTAACAAC	Chr2:65,601,925-65,601,947
				do291	GCAAATCTTGTTGTACTAACAAA	Chr2:65,601,925-65,601,947
				do292	GAAGAGATTGAATGGGATTTGCAG	Chr2:65,601,975-65,601,952
ps x maj	KASP	Fla_set 37		do317	TGAGGTAAGAATGTGCACTGAG	Chr2:29,734,621-29,734,642
				do318	TGAGGTAAGAATGTGCACTGAT	Chr2:29,734,621-29,734,642
				do319	TATGAATCTTTATCATGACCAAC	Chr2:29,734,673-29,734,651
	KASP	Fla_set 39		do323	CCATTACTTGAATCAAAGTAAGA	Chr2:64,037,674-64,037,652
				do324	CCATTACTTGAATCAAAGTAAGG	Chr2:64,037,674-64,037,652
				do325	GTATTCTTCAGCATTGCTTACTC	Chr2:64,037,614-64,037,636
	KASP	Fla_set 23		details as above		
	KASP	Fla_set 31		details as above		
	KASP	Fla_set 36		details as above		
	KASP	Fla_set 33		details as above		
	KASP	Fla_set 38		details as above		
	KASP	Fla_set 42		details as above		
	KASP	Fla_set 43		details as above		

Table S3. Loci and Alleles used in this study.

locus	centre	width	p_0	p_1
CREMOSA	13.86 (13.72 – 13.97)	3.01 (2.73 – 3.43)	0.05 (0.038 - 0.061)	0.91 (0.884 - 0.921)
FLAVIA down	13.90 (13.78 – 14.10)	3.00 (2.62 – 3.58)	0.03 (0.011 - 0.042)	0.94 (0.913 - 0.967)
FLAVIA up	10.57 (10.23 – 10.95)	9.05 (8.46 – 10.07)	0.11 (0.089 - 0.184)	0.99 (0.991 - 0.999)
SULFUREA	13.55 (13.55 – 13.56)	9.34 (9.34 – 12.50)	0.32 (0.318 - 0.321)	0.93 (0.929 - 0.939)
RUBIA	11.91 (11.46 – 12.43)	7.57 (5.97 – 9.11)	0.13 (0.063 - 0.225)	0.95 (0.898 - 0.968)
ROSEA	13.21 (13.14 – 13.26)	0.88 (0.77 – 1.02)	0.11 (0.09 - 0.117)	0.99 (0.976 - 0.991)

Geographic cline property estimates from symmetrical sigmoid cline fitting 5-parameter model for six biallelic SNP loci linked to *ROSEA* (ros_assembly_543443), *SULFUREA* (s91_39699) and *FLAVIA* (s316_93292 and s316_257789), as well as the other divergent loci in recently described colour genes (*RUBIA* - s261_720757 and *CREMOSA* - s1187_290152). The best fitting set of parameters are shown, describing the best cline fit with 95% confidence intervals (in parentheses) and include c = cline centre, w = cline width (1/gradient), p_0 = allele frequency at the asymptote in the west (*A.m.m.* var. *striatum* parental allele frequency) and p_1 = allele frequency at the asymptote in the east (*A.m.m.* var. *pseudomajus* parental allele frequency). Parameter fits for $F_{ST} = var(p)/\bar{p}(1-\bar{p})$ were conducted but not shown here.
